# Comprehensive DNA methylation profiling of COVID-19 and hepatocellular carcinoma to identify common pathogenesis and potential therapeutic targets

**DOI:** 10.1186/s13148-023-01515-8

**Published:** 2023-06-12

**Authors:** Huiyan Luo, Jixin Chen, Qiyin Jiang, Yifan Yu, Miaolun Yang, Yuehua Luo, Xiongwen Wang

**Affiliations:** 1 The First Clinical Medical College, Guangzhou University of Chinese Medicine, Guangzhou, China; 2 Lingnan Medical Research Center, Guangzhou University of Chinese Medicine, Guangzhou, China; 3 The Second Clinical College, Guangzhou University of Chinese Medicine, Guangzhou, China; 4 Department of Oncology, The First Affiliated Hospital of Guangzhou University of Chinese Medicine, Guangzhou, China

**Keywords:** Hepatocellular carcinoma (HCC), COVID-19, SARS-CoV-2, DNA methylation, Hub genes, Prognosis

## Abstract

**Background & aims:**

The effects of SARS-CoV-2 infection can be more complex and severe in patients with hepatocellular carcinoma (HCC) as compared to other cancers. This is due to several factors, including pre-existing conditions such as viral hepatitis and cirrhosis, which are commonly associated with HCC.

**Methods:**

We conducted an analysis of epigenomics in SARS-CoV-2 infection and HCC patients, and identified common pathogenic mechanisms using weighted gene co-expression network analysis (WGCNA) and other analyses. Hub genes were identified and analyzed using LASSO regression. Additionally, drug candidates and their binding modes to key macromolecular targets of COVID-19 were identified using molecular docking.

**Results:**

The epigenomic analysis of the relationship between SARS-CoV-2 infection and HCC patients revealed that the co-pathogenesis was closely linked to immune response, particularly T cell differentiation, regulation of T cell activation and monocyte differentiation. Further analysis indicated that CD4^+^ T cells and monocytes play essential roles in the immunoreaction triggered by both conditions. The expression levels of hub genes MYLK2, FAM83D, STC2, CCDC112, EPHX4 and MMP1 were strongly correlated with SARS-CoV-2 infection and the prognosis of HCC patients. In our study, mefloquine and thioridazine were identified as potential therapeutic agents for COVID-19 in combined with HCC.

**Conclusions:**

In this research, we conducted an epigenomics analysis to identify common pathogenetic processes between SARS-CoV-2 infection and HCC patients, providing new insights into the pathogenesis and treatment of HCC patients infected with SARS-CoV-2.

**Supplementary Information:**

The online version contains supplementary material available at 10.1186/s13148-023-01515-8.

## Introduction

Coronavirus disease 2019(COVID-19), caused by the severe acute respiratory syndrome coronavirus 2 (SARS-CoV-2) virus, has become a global pandemic since its discovery in late 2019, posing a significant public health threat worldwide [1, 2]. As of November 25, 2022, the World Health Organization (WHO) has reported over 636.4 million diagnosed cases and 6.6 million deaths due to COVID-19 [3]. The virus has also given rise to different variants, such as Alpha, Beta, Delta, Omicron, and others, posing continuous challenges to the population [4]. According to global phylogenetic estimation, SARS-CoV-2 has been undergoing a slow mutation of two mutations per month [5]. However, the genome of SARS-CoV-2 is relatively stable compared to other RNA viruses such as HCV and HIV, which offers the possibility of developing antibody drugs and small molecule drugs against COVID-19 [6]. Patients with COVID-19 commonly present with respiratory symptoms such as fever, cough and shortness of breath, but may also experience gastrointestinal symptoms such as nausea, loss of appetite and diarrhoea [7]. However, COVID-19 can affect organs beyond the respiratory system. Studies have shown that 10–65% of COVID-19 may have abnormal liver biochemical parameters, such as elevated liver enzyme levels. This may be due to systemic inflammatory reaction, hypoxia–ischemia reperfusion and drug-induced liver injury. Additionally, SARS-CoV-2 can infect hepatocytes, causing direct liver injury and triggering immunopathology in the liver [8]. Elevated transaminases and bilirubin have been reported to be at least twice as high in severe COVID-19 patients as compared to those with mild to moderate disease [9]. Patients with underlying malignancies are more vulnerable to SARS-CoV-2 infection and have a higher mortality rate [10]. A multi-center study of COVID-19 patients with chronic liver disease (CLD), showed that all-cause mortality rate from hepatocellular carcinoma (HCC) was approximately seven-times higher compared to patients without HCC. Another cohort study by Kim indicated that HCC was an independent risk factor for higher overall mortality in COVID-19 patients (hazard ratio [HR] 3.31 [1.53–7.16]) [11]. Further research by Li et al. confirmed that HCC patients infected with SARS-CoV-2 had a poorer prognosis and those who underwent surgery for liver cancer were at a higher risk of contracting the virus due to compromised immunity and other adverse health conditions [12, 13].

The severity of COVID-19 disease is well-known to be correlated with a various risk factors, including advanced age, cardiovascular disease, diabetes, obesity and immunocompromised conditions [10, 14]. Despite clinical factors being recognized as risk factors, they may not fully explain the variability in COVID-19 disease severity between individuals. There have been reports of severe cases among young individuals and in family gatherings, indicating that host genetic predisposition may also play a role in disease severity [15]. It has also been reported that host genetic factors are strongly associated with susceptibility to and severity of disease infection, and analysis of the human genetic data can provide insights into the mechanisms of viral infection and identify potential drug targets [16].

Epigenetics refers to the expression of stable heritable phenotypes that are influenced by environmental and metabolic factors. It plays a crucial role in the formation and evolution of many common diseases, often without altering the DNA nucleotide sequence but by modifying the chromatin structure. This unique feature makes it possible for rapid response to changes in the environment and is reversible. Viruses belonging to the coronavirus and influenza virus families typically do not directly alter host genetic sequence, but they can establish infection and spread by affecting the host epigenome. By disrupting the host’s immune reaction initiation through modulation of the epigenetic regulatory network, viruses can impact the susceptibility of older individuals to infection, especially in older population through the interaction between viral S protein, ACE2 and DPP-4 [17]. Recent evidence suggests that epigenetic drugs commonly used in cancer treatment may have broad-spectrum antiviral effects and can also be employed for inflammation control [18]. Based on this evidence, it is believed that DNA methylation plays an important role in disease progression when patients with HCC are infected with SARS-CoV-2, and studying biomarkers associated with this clinical severity is of great significance. Analyzing the epigenomics of COVID-19 and HCC can potentially aid in predicting HCC patients at higher risk for poorer prognosis if infected with SARS-CoV-2, allowing for early intervention to prolong patient survival and reduce the burden on the healthcare system.

In this study, we conducted an analysis of the epigenomics of SARS-CoV-2 infection and HCC patients, attempting to identify potential common pathogenetic process between the two diseases using techniques such as the weighted gene co-expression network analysis (WGCNA). The modules of interest that were identified were then subjected to LASSO regression analysis to identify hub genes. Subsequently, the hub genes were further analyzed, and drug candidates and their binding modes to key COVID-19 macromolecular targets were identified using molecular docking and other methods. The objective of this study is to identify shared pathogenesis and potential therapeutic targets between COVID-19 and HCC through a comprehensive analysis of DNA methylation from an epigenetic perspective. This research aims to provide new insights into the pathogenesis and therapy of HCC patients who are also infected with SARS-CoV-2.

## Materials and methods

### Data source and research objects

Methylation data of COVID-19 patients were retrieved from the Gene Expression Omnibus (GEO) database. The GEO database, which is curated and maintained by the National Center for Biotechnology Information. The database contains next-generation sequencing and high-throughput functional genomic data [19]. For our study, we selected the GSE179325 dataset for COVID-19 group, among the 574 sample, 473 were positive and 101 were negative for COVID-19 (https://www.ncbi.nlm.nih.gov/geo/query/acc.cgi?acc=GSE179325). To collect data on HCC patients, we used The Cancer Genome Atlas (TCGA) database(https://portal.gdc.cancer.gov/repository). The TCGA database aims to be a comprehensive "atlas" of cancer genomic profiles, including clinical information, transcriptomic, and epigenomic data for a wide range of human cancers. We downloaded methylation data, RNA-seq data and clinical information for 371 HCC patients from TCGA database. The methylation data included 380 tumor tissues and 50 normal tissues, while the mRNA sequencing data consisted 374 tumor tissues and 50 normal tissues. We analyzed another methylation data, GSE174818, which included 102 SARS-CoV-2 infected patients and 26 non-COVID-19 patients to validation. In addition, we analyzed two validation datasets, GSE144269 and GSE214846, with relatively large sample sizes that are associated with RNA expression of HCC. GSE144269 contains a total of 140 RNA-seq samples (70 tumor tissues and paired non-tumor tissues). GSE214846 contains a total of 130 samples (65 tumor tissue samples and 65 normal paired liver tissue samples). As the GEO and TCGA databases do not contain any personal information about patients and are publicly available, our study did not require any approval from an ethics committee.

### Quality control, normalization and differential methylation position analysis

Bisulphite converted DNA samples from the GSE179325 dataset were hybridised to the Illumina Infinium MethylationEPIC Beadchip, while the TCGA dataset was hybridised to the Illumina Methylation 450 k Beadchip. Both datasets used Illumina's probe annotation for gene annotation. Methylation levels of CpG sites were quantified in terms of β values, and the downloaded matrix of β values was pre-processed and quality controlled in R (v.4.2.1) using the ChAMP package, an integrated analytical pipeline for the analysis of Illumina HumanMethylation BeadChips that can be used to filter low-quality probes, normalize data, correct for batch effects, detect differential methylation positions (DMPs) etc. [20]. We filtered probes according to the following conditions: (1) probes with p-values > 0.01; (2) probes with a bead count of less than 3 in at least 5% of samples; (3) non-CpG probes; (4) probes associated with SNPs; (5) probes with multiple hits;(6) all probes located on chromosomes X and Y. We normalized with BMIQ method(champ.norm() function) to adjust for Type II probe detection bias. The champ.DMP() function from the limma package was used to calculate the p-value for differential methylation using a linear model. P-adjust values were corrected using the Benjamini & Hochberg method, and probes with p-adjust values < 0.05 were considered DMPs. We analyzed DMPs in COVID-19 positive and negative patients and between HCC tumor tissue and normal tissue. The DMPs were classified according to their chromosome location and the feature category gene regions, including TSS1500, TSS200, 5′ UTR, 1st Exon, Body, 3′ UTR and IGR.

### Gene ontology enrichment analysis

To gain a deeper understanding of the biological functions of the methylation-driven genes, we performed Gene Ontology (GO) enrichment analysis on the differentially methylated genes(DMGs) found in the promoter regions (TSS1500, TSS200, 5′ UTR, 1st Exon) of both the COVID-19 and HCC groups. We conducted GO enrichment analysis in R using the "clusterProfiler" package, and the top ten key items of cellular components (CC), molecular functions (MF) and biological processes (BP) were visualized using the "ggplot2" package [21].

### Immune infiltration level analysis

We used EpiDISH, a reference-based cell type deconvolution algorithm, to identify specific cell types exhibiting differential DNA methylation [22, 23]. Based on the methylation β-values of CpGs, we computed the proportions of seven immune cell types (including B-cells, Natural Killer (NK) cells, CD4^+^ T-cells, CD8^+^ T-cells, Monocytes, Neutrophils and Eosinophils) in the dataset using the "EpiDISH" R package.

### The weighted gene co-expression network analysis

We used the R software package "WGCNA" to structure a co-expression network of the screened DMPs [24]. To determine the soft threshold power β, we calculated the pickSoftThreshold function based on the approximate scale-free topology formula. We then calculated an adjacency matrix, which was converted to a topological overlap matrix (TOM). Co-expression networks were constructed and we identified at least 30 DMPs in each module(minModuleSize = 30), and selected modules required for intramodular analysis based on their correlations with the immune cells matrix, calculated using the Pearson correlation method. We used the absolute values of correlations between DMPs and traits to quantify the associations of individual DMPs with specific immune cells, defining them as Gene significance (GS). Module membership (MM) was used to correlate module DMPs and methylation expression profile. Together, GS and MM quantify the correlation between the target module DMPs and immune cells, which we visualized in a scatterplot. We then extracted genetic information from the corresponding module for subsequent analysis.

### Differentially expressed gene analysis

We analyzed mRNA expression data to identify differentially expressed genes (DEGs) between HCC tumor tissue and normal tissue. We used the “edgeR” package to perform this analysis, where genes with a |log2 (fold change)|> 1 and p < 0.05 was considered to be significant DEGs [25]. To visualized the DEGs, we created volcano plots. Additionally, we used TBtools software (v1.098775) to draw heatmaps displaying the top 15 genes [26].

### Hub genes identification and the prognostic risk model construction

To avoid overfitting, we implemented least absolute shrinkage and selection operator (LASSO) regression to identify the most informative genes with the best prognostic features. Cox proportional hazard models were then constructed using the selected hub genes, and the patient's risk score was calculated using the formula: Risk score = β1 * exp hub gene1 + β2 * exp hub gene2 + … + βn * exp hub gene, where β1 to βn represents the prognostic coefficient of each hub gene, and exp hub gene represents the expression level of the respective hub gene. We separated the patients into high-risk group and low-risk group using the median risk score as a cut-off, and further analysis were performed on these two groups.

### Gene regulatory networks analysis

To identify the interactions between hub genes and microRNAs (miRNAs) as well as transcription factors (TFs), we constructed a gene regulatory network. First, we utilized two databases, TarBase and miRTarBase, to select hub gene-miRNA interactions with experimental support such as reporter gene assays, microarrays, proteomics and next-generation sequencing experiments [27, 28]. To improve the accuracy of our predictions, we selected only hub gene- miRNA interactions that were present in both the TarBase and miRTarBase. Additionally, we used the ChIP-X Enrichment Analysis 3(ChEA3) database, which contains a collection of gene set libraries from multiple sources and is a TF enrichment analysis tool, to identify the top 15 TFs that were closely related to the hub gene [29]. Finally, we utilized Cytoscape (V3.9.0) to create the gene regulatory network using data from the aforementioned networks.

### Evaluation of applicant drugs

Enrichr is an online platform that hosts a large collection of genes and libraries that can be used for enrichment analysis to discover biological knowledge [30]. The Drug Signature Database (DSigDB), a component of Enrichr, links drugs and compounds to their target genes, enabling repurposing and translational studies [31]. We utilized Enrichr to access DSigDB to identify candidate drugs and compounds that were enriched with hub genes for further analysis.

### Molecular docking simulation

Molecular docking is a key tool for drug discovery and development of new drugs that predicts the bond conformation and orientation of small molecule ligand within macromolecular targets at protein binding sites. QuickVina-W is a high-precision, inter-process spatio-temporal integration method for virtually screening of large ligands libraries [32]. We used QuickVina-W for "virtual screening" to predict the binding sites and conformation of drug candidates with key macromolecular targets of COVID-19. To identify these key macromolecular targets, we conducted an extensive literature search and selected PLpro, Mpro, RdRp, S protein. Protein crystal structures of these targets were downloaded from RCSB Protein Data Bank (PDB ID: PLpro(4OVZ), Mpro(6LU7), RdRp(6NUS), S protein(6VSB)), while structures of drug candidates were obtained from the PubChem database [33, 34]. The virtual docking was performed with QuickVina-W (exhaustiveness = 64) and binding sites interactions were analyzed with the Protein–Ligand Interaction Profiler. Docking results were visualized with PyMOL (v 2.4.0) [35].

### Cell culture

The human HCC cells Huh7 and hepatocyte cell L-02, which were purchased from Chinese Type Culture Collection (CTCC, Shanghai, China), were routinely cultured in Dulbecco’s modified Eagle's medium–high glucose (Catalog: PM150210, Procell) containing 10% fetal bovine serum (Catalog:HN-FBS-50, HAKATA) and 1% penicillin/streptomycin (Catalog: C125C5, NCM Biotech). All cells were cultured in a humidified incubator at 37 °C and 5% CO_2_.

### Real-time RT-PCR

Total RNA was extracted by AG RNA Pro Reagent (Catalog: AG21101, Accurate Biology). According to the kit instructions, cDNA was reverse transcription by Evo-M-MLV RT Premix (Catalog: AG11706, Accurate Biology). The qRT-PCR reactions were prepared with SYBR Green Premix Pro TaqHS qPCR Kit (Catalog: AG11739, Accurate Biology). The qPCR primers were shown in Additional file 11: Table S2.

### Statistical analysis

Most of the statistical analysis and plot production were constructed using by R (v.4.2.1), including differential methylation analysis, WGCNA, expression analysis, survival analysis, etc. Part of the statistical analyses and graphs were generated using online websites and bioinformatics tools such as the the TarBase, miRTarBase, ChEA3, DSigDB database and the TBtools software. The gene regulatory networks were constructed by Cytoscape version 3.9.0. Molecular docking simulation was performed using QuickVina-W and visualized with PyMOL. The 2–ΔΔ Ct method was used to analyze the RNA expression data of hub genes from qPCR experiments. The experiment was repeated 3 times, independently. GraphPad Prism (v.7.00) was used for graphing and statistics. The statistical method selected was the t-tests. A p-value less than 0.05 was considered to be a statistically significant.

## Results

### Study design and characteristics of participants

**Fig. 1:**
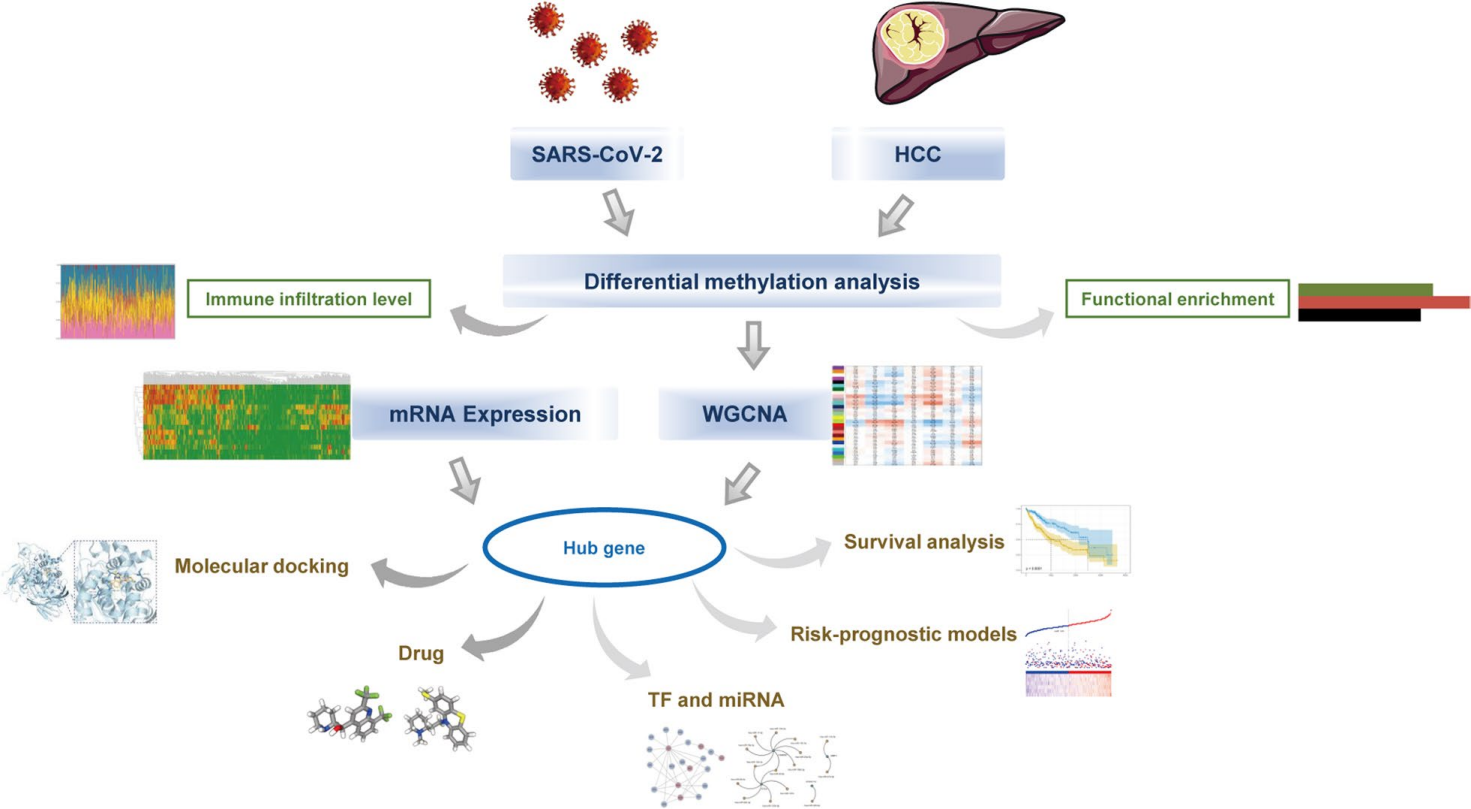
The flow chart of this comprehensive profiling

Summary of the research is presented in Fig. 1. Clinical characteristics of patients with COVID-19 and HCC, including age, gender, and disease status are displayed in Table 1. The study begins by analyzing the epigenetic correlation between SARS-CoV-2 infection and HCC, followed by identifying the common pathogenesis between the two diseases. To identify key modules, a WGCNA network was constructed, and DMP in the key modules was analyzed jointly with the mRNA expression data of HCC. The hub genes were then screened using LASSO regression, and a prognostic risk model was constructed. To further explain the common pathogenesis between SARS-CoV-2 infection and HCC and to offering new insights for treatment, gene regulatory network analysis of hub genes and was performed, and drug candidates for virtual docking were identified.

**Table 1:** Characteristics of patients from SARS-CoV-2 infection and HCC

Characteristic	HCC n = 371	Characteristic	COVID-19 n = 574
Gender, n (%)		Gender, n (%)	
Male	250(67.39)	Male	287(50.00)
Female	121(32.61)	Female	287(50.00)
Age, year(mean ± SD)	59.44 ± 13.52	Age, year(mean ± SD)	67.00 ± 17.26
Grade, n (%)		Disease state, n (%)	
1	55(14.82)	Severe	113(19.69)
2	177(47.71)	Mild	360(62.72)
3	122(32.88)	Negative	101(17.60)
4	12(3.23)		
Unknown	5(1.35)		
Vital status, n (%)			
Alive	282(76.01)		
Dead	89(23.99)		

SD, standard deviation

### Determining the epigenetic relevance between SARS-CoV-2 infection and HCC

**Fig. 2:**
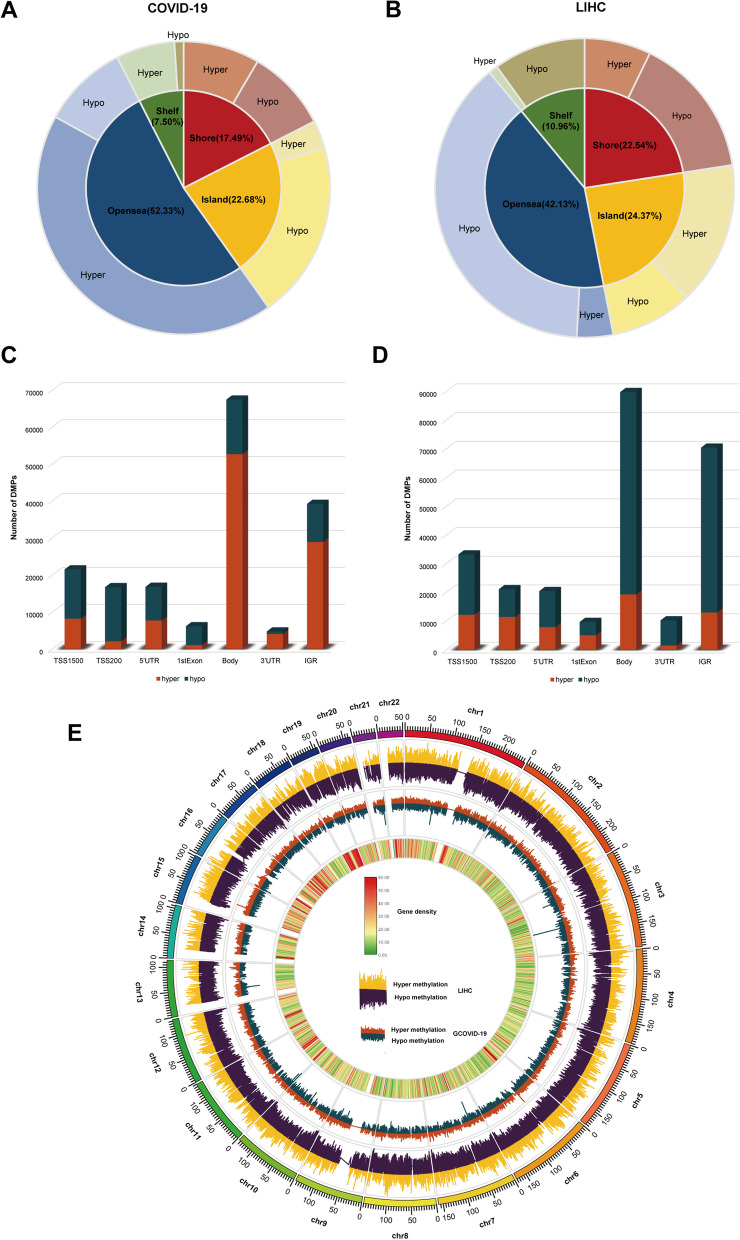
Differential methylation positions (DMPs) identified in SARS-CoV-2 infection and HCC. **A**–**B** The location of the DMPs relative to CpG islands in SARS-CoV-2 infection (**A**) and HCC (**B**). **C**–**D** The location of the DMPs relative to gene regions in SARS-CoV-2 infection (**C**) and HCC (**D**). **E** Combined analysis of DMPs located in the promoter region in SARS-CoV-2 infection and HCC

**Fig. 3:**
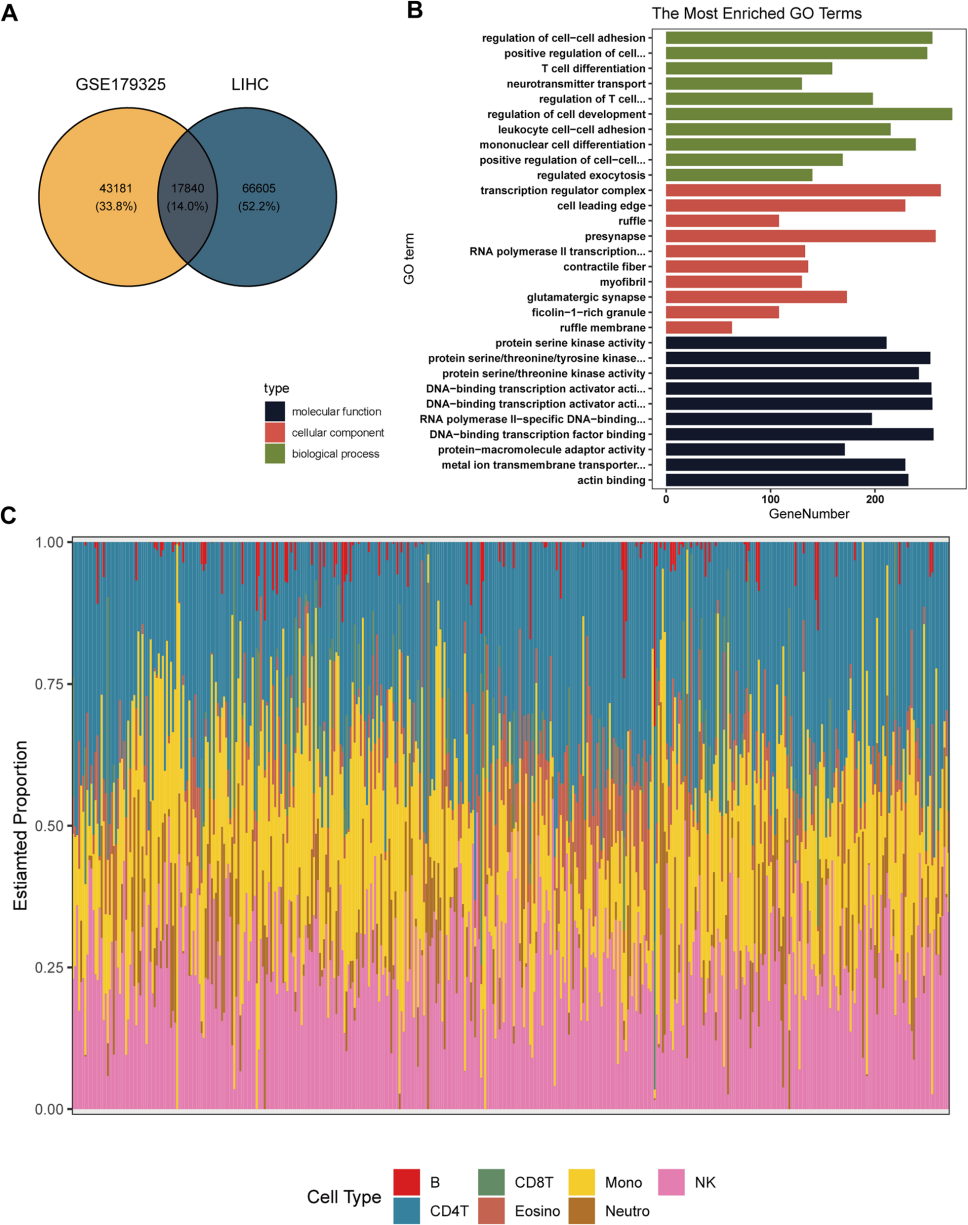
Enrichment analysis and immune infiltration level analysis. **A** Number of DMPs located in the promoter region in SARS-CoV-2 infection and HCC. **B** Results of GO enrichment analysis of DMPs present in SARS-CoV-2 infection and HCC. **C** Evaluation of the seven different kinds of immune cells in HCC samples

After the imputation of missing values, 2498 non-CpG probes and 34,851 SNP-related probes were filtered from the COVID-19 dataset. In the HCC dataset, 3156 non-CpG probes, 59,901 SNP-related probes, 11 multiple hits and 10,028 probes located in the X and Y chromosomes were removed. We furthered analyzed the filtered probes and found a total of 17,306 DMPs between COVID-19 positive and negative samples. Of these DMPs, 52.33% were located in Opensea, 22.68% in Island, 17.49% in Shore and 7.50% in Shelf (Fig. 2A). In the COVID positive samples, there were 105,754 hypermethylated CpG and 67,952 hypomethylated CpG, of which 35.13% were located in the promoter region (Fig. 2C). For the HCC dataset, we found a total of 254,815 DMPs between HCC tumor tissue and normal tissue. Of these DMPs, 42.13% were located in Opensea, 24.37% in Island, 22.54% in Shore and 10.96% in Shelf (Fig. 2B). In the tumor tissue, there were 70,390 hypermethylated CpG and 184,425 hypomethylated CpG, of which 33.14% were located in the promoter region (Fig. 2D). To examine methylation changes across the genome, we performed a conjoint analysis, and the results are shown in Fig. 2E. We selected 17,840 DMPs (Fig. 3A) that were present in both COVID-19 and HCC datasets and located in the promoter region for GO enrichment analysis. The results suggested a close involvement of main BP in immune response such as T cell differentiation, regulation of T cell activation and monocyte differentiation (Fig. 3B). We then calculated the abundance of seven immune cells in the HCC dataset (Fig. 3C) and the abundance of immune cells in the COVID-19 dataset (Additional file 1: Figure S1).

### Co-expression network analysis and key module identification

**Fig. 4:**
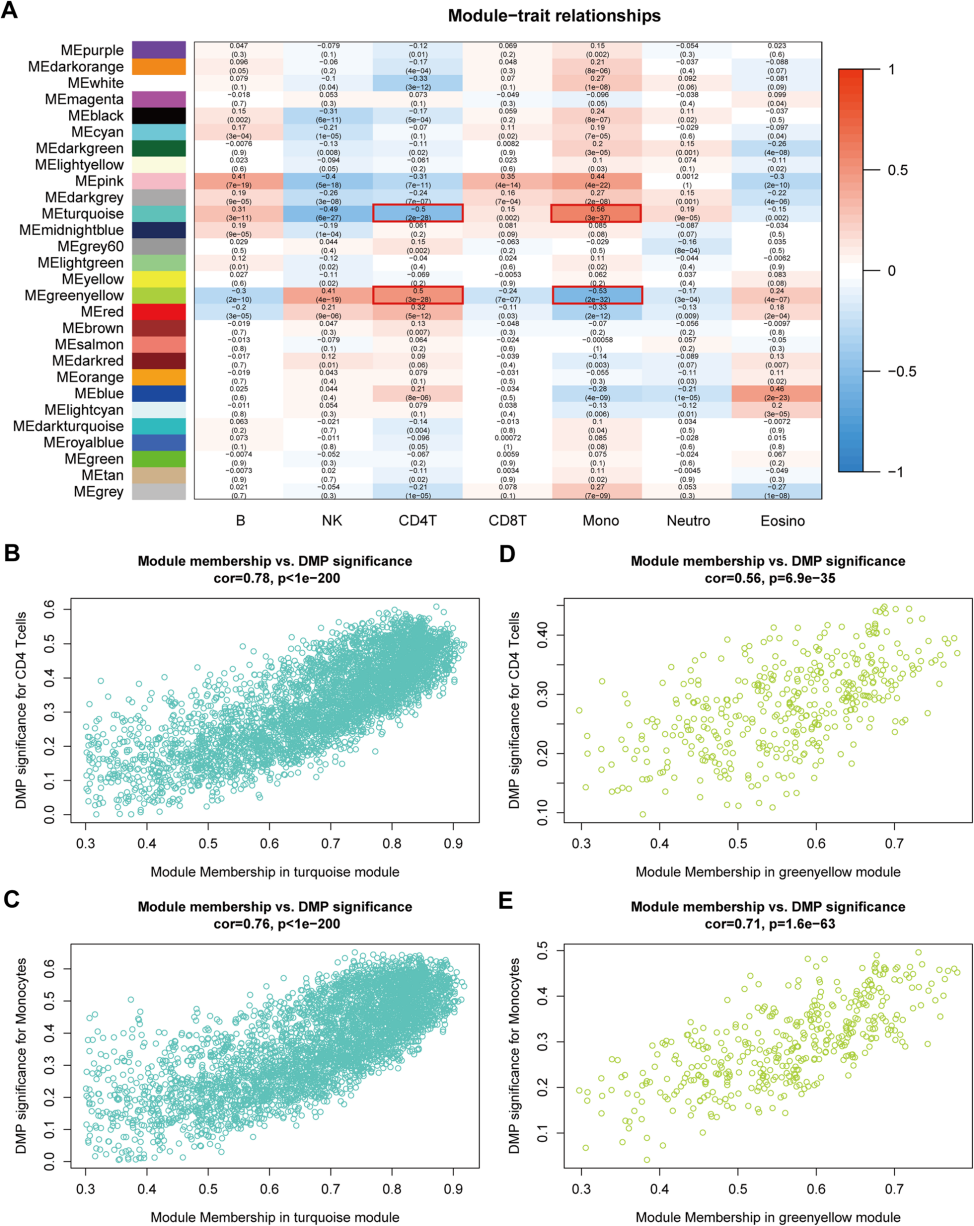
The weighted gene co-expression network analysis. **A** Correlation analysis of module and immune cell expression abundance. **B**–**C** Correlation analysis of turquoise color modules with CD4^+^ T cells (**B**) and monocytes (**C**). **D**–**E** Correlation analysis of greenyellow color modules with CD4^+^ T cells (**D**) and monocytes (**E**)

We constructed a weighted co-expression network with a soft threshold of β = 3 (Additional file 2: Figure S2) and correlated it with modules with expression of immune cell abundance. As shown in Fig. 4A, our results revealed that both the turquoise color module (CD4^+^ T cells: r = -0.5, p = 2e-28; monocytes: r = 0.56, p = 3e-37) and greenyellow module (CD4^+^ T cells: r = 0.5, p = 3e-28; monocytes: r = -0.53, p = 2e-32) were closely associated with CD4^+^ T cells and monocytes, making them modules of interest for further analysis. The high correlation between GS (y-axis) and MM (x-axis) was confirmed by scatterplot results (CD4^+^ T cells: turquoise, cor = 0.78, p < 1e-200; greenyellow, cor = 0.56, p = 6.9e−35. Monocytes: turquoise, cor = 0.76, p < 1e-200; greenyellow, cor = 0.71, p = 1.6e−63) (Fig. 4B–E).

### Integration of epigenomic and transcriptomic data

**Fig. 5:**
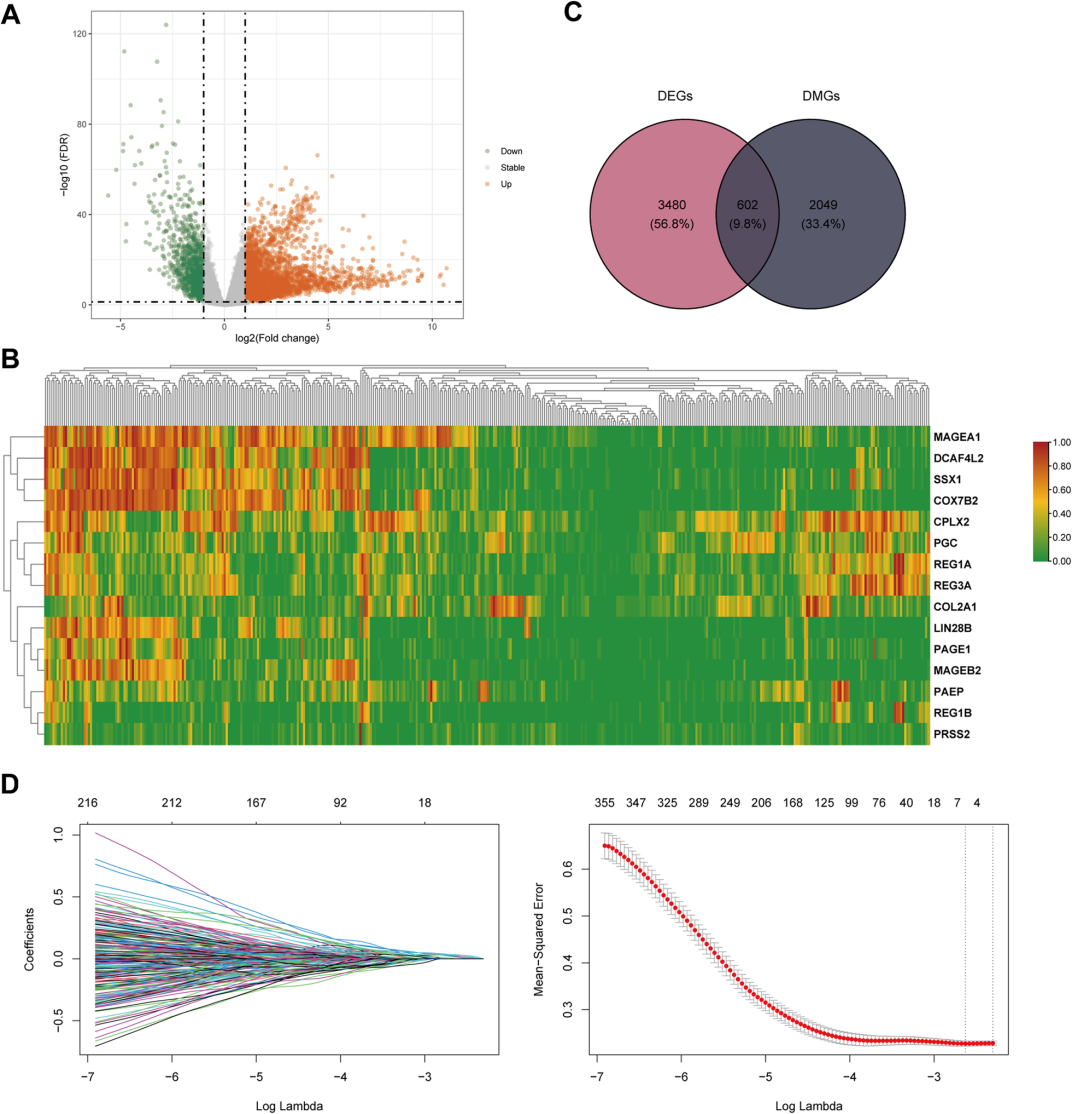
Identification of hub genes. **A** Genes with differential mRNA expression in HCC tumor tissue and normal tissue. **B** The top 15 most differentially expressed genes. **C** Identification of genes with both differential methylation regulation and mRNA expression. **D** The LASSO coefficient spectrum of the 604 mRNAs. **E** The relationship between the mean squared error curve and versus log (λ), using the minimum criteria and 1-standard error to plot the dashed vertical line at the best value

To systematically determine the molecular mechanisms underlying the pathogenesis of both SARS-CoV-2 infection and HCC, we conducted an analysis of RNA-seq data from HCC. Our results revealed there were 4082 DEGs between HCC tumor tissue and normal tissue, with 3062 genes up-regulated and 1020 genes down-regulated (Fig. 5A). The top 15 most differentially expressed genes were shown in Fig. 5B. Next, we performed a co-analysis DEGs with DMGs in the modules of interest, and identified 602 genes that exhibited both differential methylation regulation and mRNA expression for subsequent analysis (Fig. 5C). To determine the relationship between these screened genes and HCC prognosis, we used LASSO regression to identify hub genes. As log lambda (an adjustment parameter) was varied, the relative coefficients of certain genes were compressed and gradually towards zero. We selected gene with non-zero coefficients at the best lambda value (lambda.min) and obtained a total of six hub genes (Fig. 5D, E): MYLK2, FAM83D, STC2, CCDC112, EPHX4 and MMP1.

### Analysis of hub genes

**Fig. 6:**
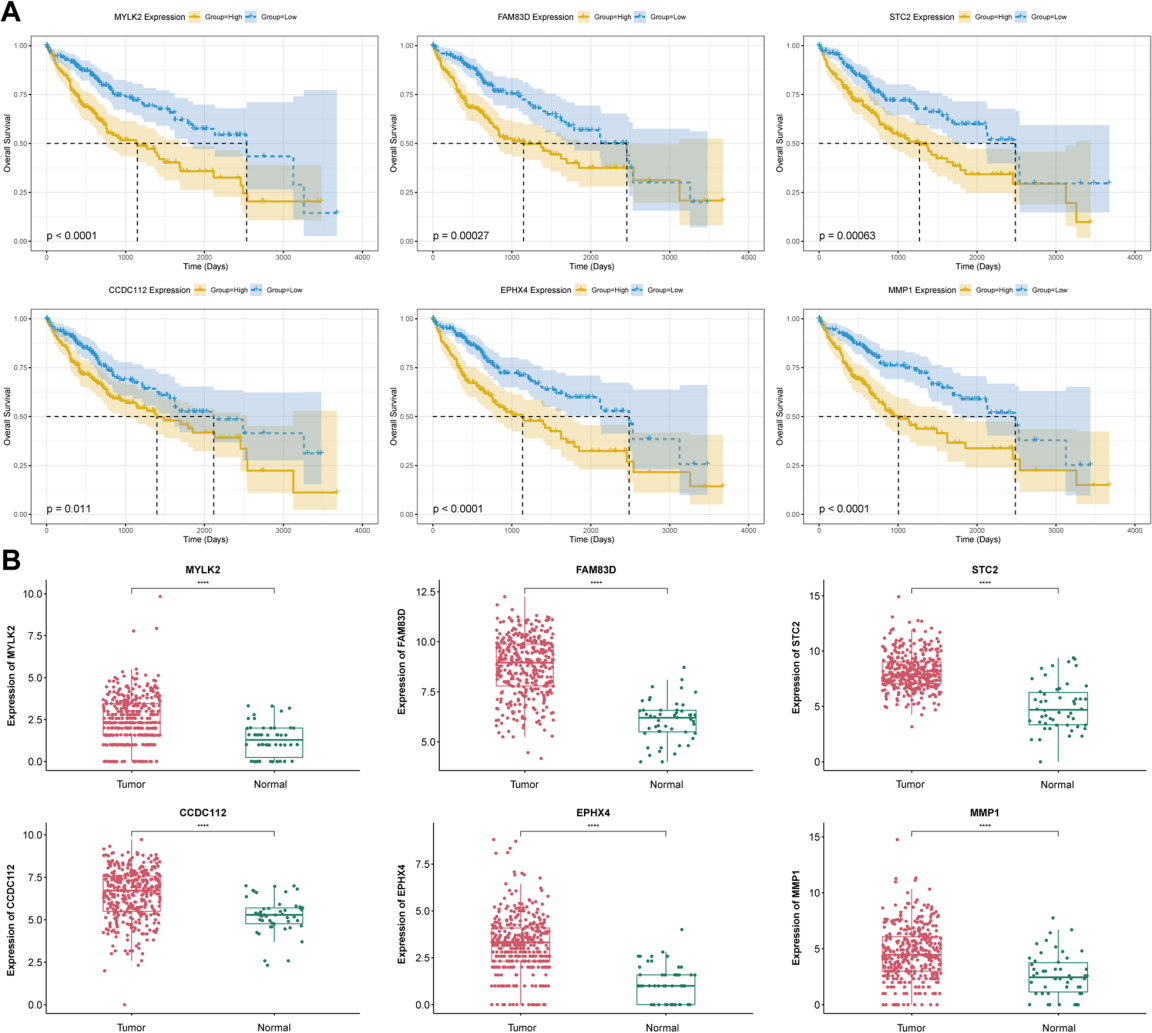
Analysis of hub genes. **A** Survival analysis of hub genes. **B** The expression of hub genes in tumor tissue and normal tissues (****p < 0.0001)

To investigate the impact and significance of hub genes on the prognosis of HCC patients, we conducted a survival analysis using the overall survival (OS) time of patients. The relationship between the expression of key genes and survival rates was assessed using Kaplan–Meier. The results revealed a strong correlation between expression of six hub genes and patients' OS, with patients having low expression levels exhibiting a longer survival time (Fig. 6A). Furthermore, we compared the expression levels of six hub genes in HCC tumor tissue with those in normal tissue. Our analysis demonstrated that MYLK2, FAM83D, STC2, CCDC112, EPHX4 and MMP1 were differentially expressed between tumor and normal tissues, with higher expression in tumor tissues than in normal tissues (p < 0.0001) (Fig. 6B). To strengthen make our results, we added validation datasets to verify our findings. We analyzed another dataset (GSE174818) of COVID-19 patients. We compared the DMPs between the SARS-CoV-2 infected patients and the non-COVID-19 patients. The results showed that there were 60,972 DMPs between the COVID-19 positive and negative samples, with 58.64% located in Opensea, 11.49% in Island, 18.39% in Shore, and 11.48% in Shelf. Compared with the negative samples, there were 55,655 highly methylated CpG sites and 5317 hypomethylated CpG sites in the COVID-19 positive samples, with 22.22% located in promoter regions (Additional file 5: Fig. S5). In the differential methylation probes, six hub genes were identified, including MYLK2 (cg05152503, cg10194632, cg18034859, cg08726417, cg15736167), FAM83D (cg06163215, cg15195292), STC2 (cg27123016, cg08839053), CCDC112 (cg11745506, cg03317980, cg23403750), EPHX4 (cg15156367), and MMP1 (cg08451044). We compared the RNA expression of six hub genes between tumor tissues and normal tissues in GSE144269 and GSE214846, and the results suggest that MYLK2, FAM83D, STC2, CCDC112, EPHX4, and MMP1 are differentially expressed between tumor tissues and normal tissues, with higher expression in tumor tissues. We also performed experimental verification of RNA expression levels of hub genes in L-02 hepatocyte cell and human HCC cells (Huh7), and the results were consistent with our analysis (Additional file 6: Fig. S6).

### Construction of a risk prognostic model and correlation analysis

**Fig. 7:**
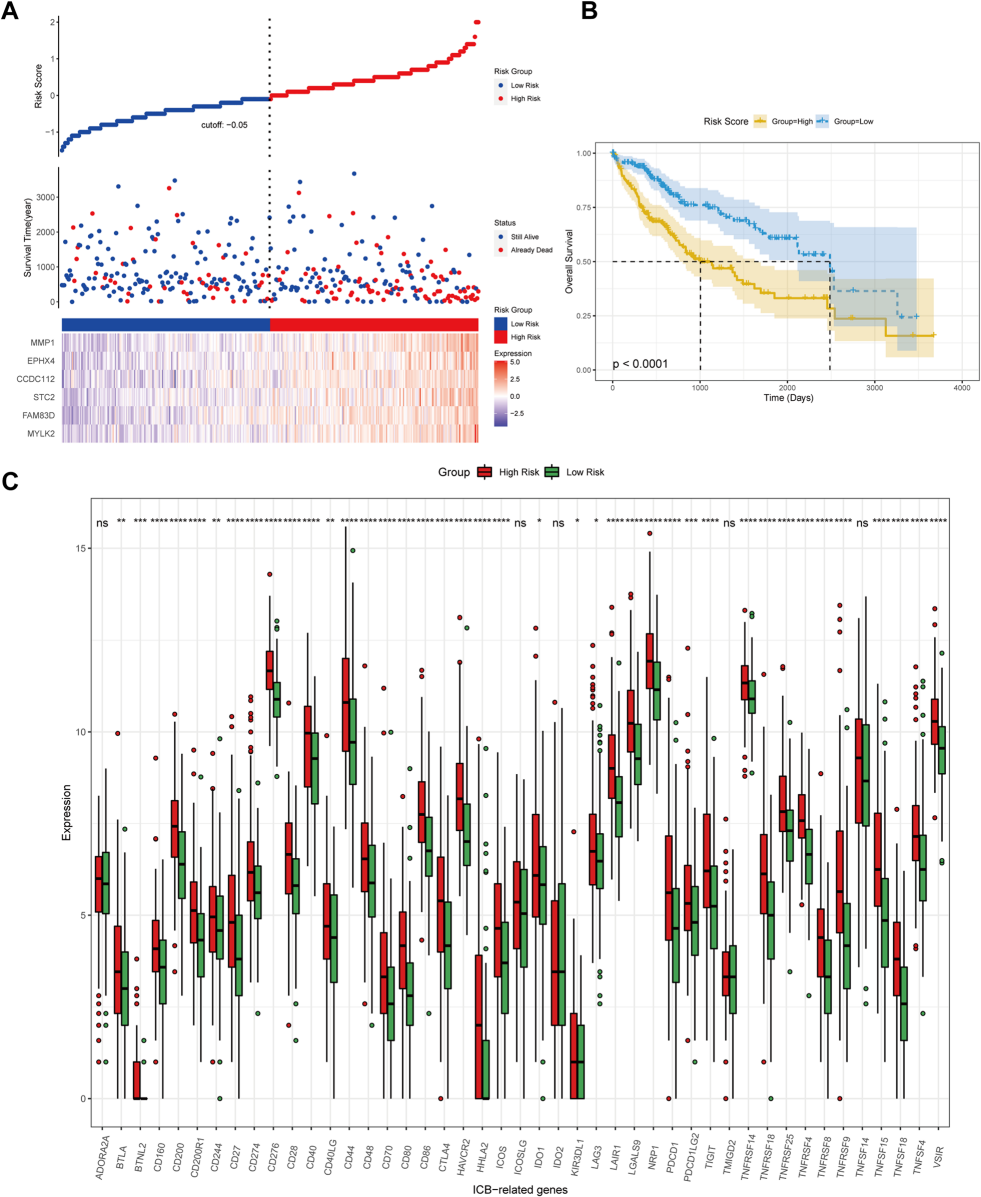
Construction of risk-prognostic models. **A** The risk prognostic model based on hub genes. (From top to bottom, the risk score map, survival status, and gene expression heatmap of different risk groups were shown). **B** Survival analysis of the association between the risk score and OS. **C** The expression of ICB-related genes in different risk score groups (*p < 0.05; **p < 0.01; ***p < 0.001; ****p < 0.0001)

We constructed a cox proportional hazard regression models using the hub genes and calculated risk scores based on gene expression values and corresponding risk coefficients (Fig. 7A). The risk score formula is as follows: Risk score = 0.12439*MYLK2 expression + 0.15204*FAM83D expression + 0.11755*STC2 expression + 0.05791*CCDC112 expression + 0.01244*EPHX4 expression + 0.10310*MMP1 expression. Using the median as cutoff value, we stratified patients into high-risk and low-risk groups and performed a survival analysis, with results revealing the low-risk group have a significantly better prognosis (Fig. 7B). In line with significant advances in immune checkpoint blockade (ICB) in the cure of tumors, we further reviewed multiple literature and selected 45 common ICB related genes for analysis. Our results showed that the expression levels of most ICB-related genes were significantly higher in the high-risk group than in the low-risk group (Fig. 7C). Furthermore, the level of ICB expression in most patients was positively correlated with risk score (Additional file 7 and Additional file 8: Figs. S7, S8).

### Analysis of gene regulatory networks

**Fig. 8:**
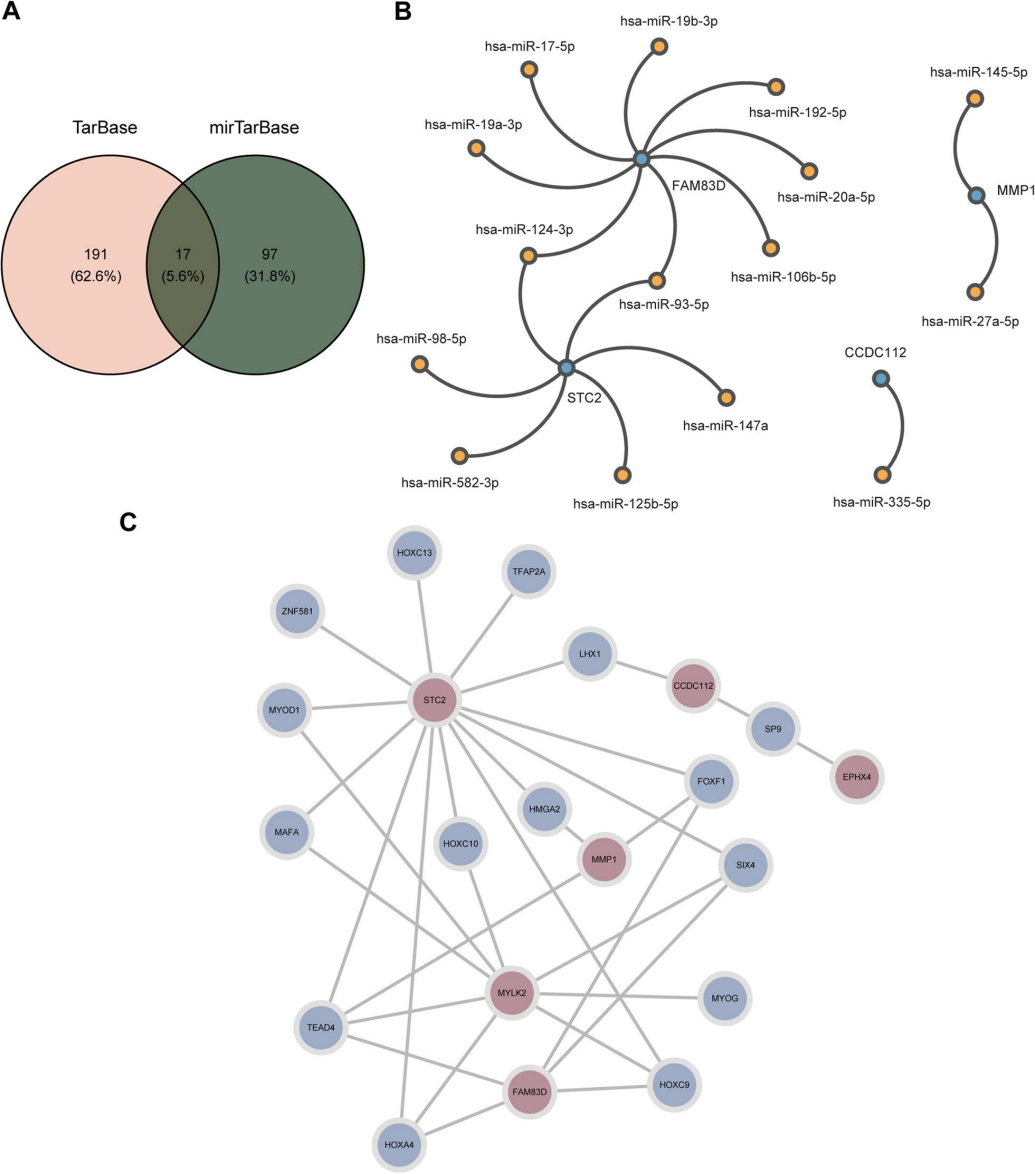
Gene regulatory networks analysis. **A** Identification of miRNAs present in both TarBase and mirTarBase. **B** Hub genes-miRNA interaction network. **C** TFs-hub genes interaction network

To gain form a deeper understanding of the regulatory mechanism of gene expression, we conducted gene regulatory network analysis on hub genes and identified their interactions networks with miRNA and TF. We retrieved 191 hub gene-miRNA action pairs from TarBase and 97 pairs from mirTarBase. Among them, 17 hub gene-miRNA pairs were present in both databases (Fig. 8A), and we constructed a regulatory network (Fig. 8B) based on these pairs. The binding sites between the hub gene-miRNAs were depicted in Additional file 4: Fig. S4. In the interaction analysis of hub genes and TFs, we identified the top 15 predicted TFs, and their predicted TF rankings is shown in Additional file 9: Fig. S9. We also visualized the network of interactions between the hub genes and TFs (Fig. 8C).

### Identification of drug candidates and virtual screening

**Fig. 9:**
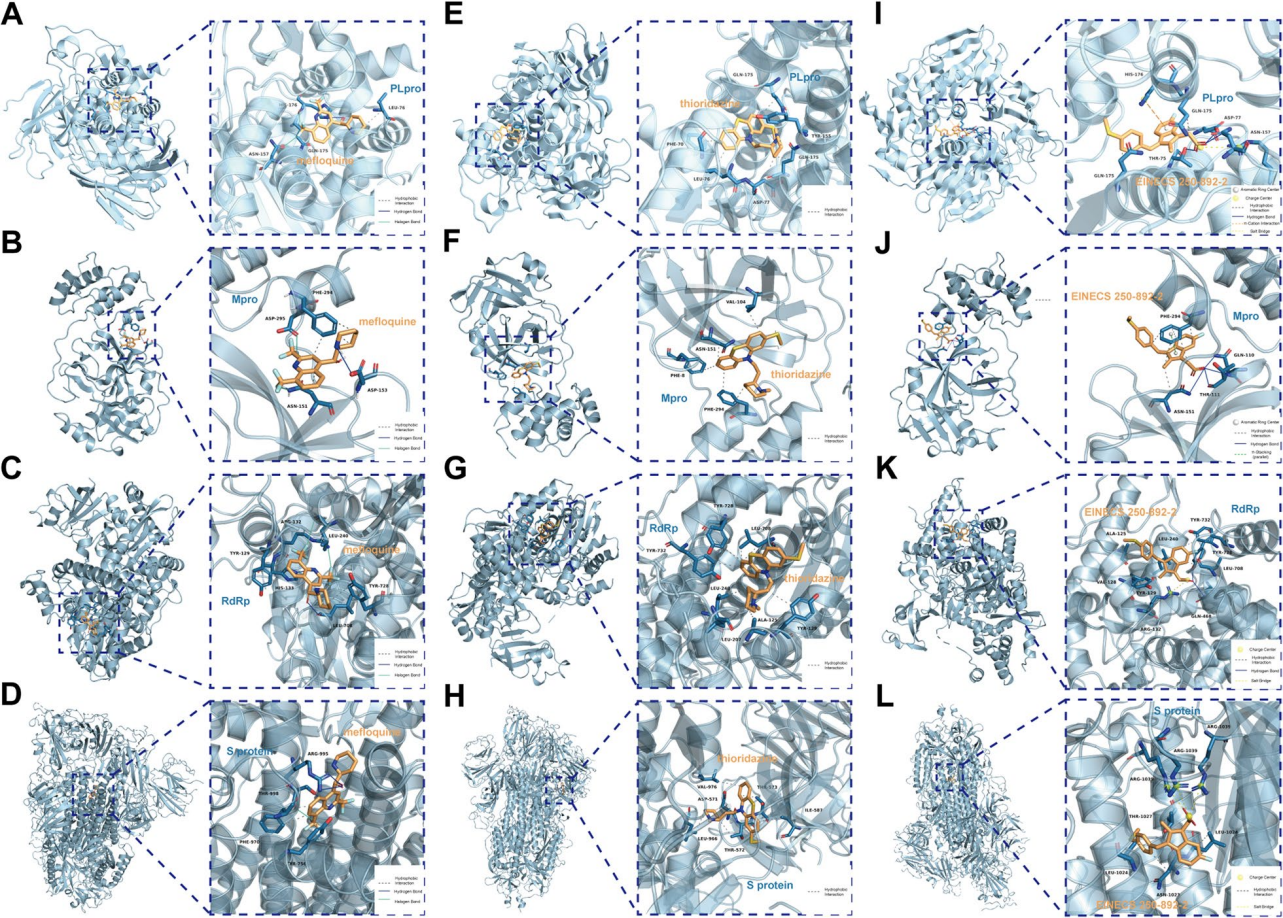
Molecular docking patterns. **A**–**D** Molecular docking patterns for mefloquine with the PLpro (**A**), Mpro (**B**), RdRp (**C**), S protein (**D**). **E**–**H** Molecular docking patterns for thioridazine with the PLpro (**E**), Mpro (**F**), RdRp (**G**), S protein (**H**). **I**–**L** Molecular docking patterns for EINECS 250-892-2 with the PLpro (**I**), Mpro (**J**), RdRp (**K**), S protein (**L**)

To identify drug molecules that can potentially regulate the hub genes, we utilized DSigDB and extracted the top five compounds based on their p-values (Table2), namely lamivudine, mefloquine, hydroquinone, thioridazine, and EINECS 250-892-2. To further investigate the potential of these compounds to target SARS-CoC-2, we perform virtual docking on these potential compounds with four key macromolecular targets of COVID-19 (PLpro, Mpro, RdRp, S protein). We analyzed the binding modes between the compounds and the macromolecular targets and calculated their binding energies. A negative binding energy indicates spontaneous chemical reactions, and the magnitude of the negative value corresponds to the affinity of the interaction between the ligand and the macromolecule. We also analyzed the interaction of amino acid residues on drug candidates with macromolecules (Table3). Finally, we identified three drug candidates with the lowest binding energy and presented their binding modes to key macromolecular targets (Fig. 9).

**Table 2:** Top 10 candidate drugs that may regulate hub genes

Drug	Molecular formula	Structure
Lamivudine	C_8_H_11_N_3_O_3_S	
Mefloquine	C_17_H_16_F_6_N_2_O	
Hydroquinone	C_6_H_6_O_2_	
Thioridazine	C_21_H_26_N_2_S_2_	
EINECS 250-892-2	C_20_H_17_FO_2_S	
Vinblastine	C_46_H_58_N_4_O_9_	
ML-9	C_15_H_18_C_l2_N_2_O_2_S	
Ilomastat	C_20_H_28_N_4_O_4_	
Aminolevulinic acid	C_5_H_9_NO_3_	
MG-262	C_25_H_42_BN_3_O_6_

**Table 3:** The binding sites and energies for candidate drugs were evaluated through molecular docking

Drug targets	Amino acid	Binding energy (kcal/mol)
**PLpro**		
Lamivudine	ARG-167, MET-207, ASP-165, THR-169, HIS-172, LYS-158	− 7.1
Mefloquine	HIS-176, ASN-157, GLN-175, LEU-76	− 9.5
Hydroquinone	GLU-78, LEU-81, PRO-60, ALA-69, PHE-80	− 5.4
Thioridazine	PHE-70, LEU-76, ASP-77, GLN-175, TYR-155, GLN-175	− 9.0
EINECS 250-892-2	THR-75, ASP-77, GLN-175, HIS-176, ARG-83, ASN-157	− 9.1
**Mpro**		
Lamivudine	LYS-102, SER-158, ASN-151, THR-111, PHE-294, GLN-110	− 5.6
Mefloquine	ASP-153, ASN-151, ASP-295, PHE-294	− 7.2
Hydroquinone	GLU-270, LEU-271, PHE-219, TRP-218, ARG-279, ASN-274	− 4.7
Thioridazine	VAL-104, ASN-151, PHE-8, PHE-294	− 6.1
EINECS 250-892-2	PHE-294, ASN-151, THR-111, GLN-110	− 7.1
**RdRp**		
Lamivudine	ALA-762, ALA-797, TRP-617	− 6.0
Mefloquine	TYR-129, HIS-133, LEU-708, TYR-728, LEU-240, ARG-132	− 8.2
Hydroquinone	ARG-305, PHE-471, GLU-474, VAL-742, ASP-738, VAL-737	− 5.0
Thioridazine	TYR-732, LEU-240, LEU-207, ALA-125, TYR-129, LEU-708, TYR-728	− 7.6
EINECS 250-892-2	ALA-125, VAL-128, TYR-129, ARG-132, GLN-468, LEU-708, TYR-728, TYR-732, LEU-240	− 7.6
**S protein**		
Lamivudine	LYS-1038, HIS-1048, SER-1037, GLN-1036, TYR-904	− 6.4
Mefloquine	ARG-995, THR-998, PHE-970, TYR-756	− 8.6
Hydroquinone	TRP-886, THR-887, LEU-894, ILE-896, ALA-713, ILE-712, ARG-1107	− 5.7
Thioridazine	VAL-976, ASP-571, LEU-966, THR-572, ILE-587, THR-573	− 8.2
EINECS 250-892-2	ARG-1039, THR-1027, LEU-1024, ASN-1023, LEU-1024	− 8.0

## Discussion

Liver cancer is currently the sixth most commonly occurring cancer worldwide. Its incidence is increasing globally, which poses a significant burden on healthcare systems and makes it a growing global health threat [36]. Current findings indicates that SARS-CoV-2 infection may lead to liver damage through various mechanisms, which may include disruptions in biochemical indicators such as aminotransferase activity and bilirubin levels [37]. The impact of SARS-CoV-2 infection on HCC patients who already suffer from chronic liver diseases such as viral hepatitis and cirrhosis is complex [38]. Research has indicated that epigenetic dysregulation following SARS-CoV-2 infection increases the risk of fatality in COVID-19 patients [39]. Abnormal epigenetic alterations can also disrupt the expression of oncogenes and tumor suppressor genes, which may promote tumor cell proliferation, metastasis, drug resistance and immune escape [40, 41]. For instance, a study conducted by Revia et al. discovered that the tumor suppressor KDM6A, a demethylase, can inhibit the progression of HCC by restructuring the epigenetic landscape and influencing mTORC1 signaling in cancer [42]. Additionally, several studies have suggested that hypermethylation of tumor suppressor genes on the CpG islands in the promoter region can lead to transcriptional silencing and gene inactivation [43].

It is widely recognized that SARS-CoV-2 infection triggers the host's immune system, activating inflammatory pathways and cytokine storms, which can result in severe outcomes, such as lung damage, acute respiratory distress syndrome (ARDS), disseminated intravascular coagulation (CID) and multi-organ failure [44]. The immune dysregulation in COVID-19 patients can be categorized into innate and adaptive immune responses. Monocytes, which are primarily associated with innate immune response, may differentiate into macrophages or dendritic cells to recruit to sites of inflammation. Following SARS-CoV-2 infection, the patients’ peripheral blood monocyte subpopulations were reduced and became more pronounced with increasing severity [45]. Numerous studies have confirmed that specific T cells phenotypes after SARS-CoV-2 infection are strongly correlated with the severity of COVID-19 patients [46–48]. Our findings are consistent with these studies, where the GO enrichment analysis showed that the primary BP involved were closely related to immune response, such as T cell differentiation, regulation of T cell activation and monocyte differentiation. HCC often results from chronic liver disease progression, as an inflammation-related tumor. Ample evidence supports the role of immune escape in tumor development and formation. In this process, a large number of macrophages, innate immune cells and adaptive immune cells come together to form a complex mirco-environment of immune tolerance [49, 50]. The results from WGCNA analysis showed that the turquoise and greenyellow modules were closely associated with CD4^+^ T cells and monocytes. We speculate that the immune responses elicited by CD4^+^ T cells and monocytes may be closely associated with disease progression when HCC patients are co-infected with SARS-CoV-2. This is in line with previous studies where Shi revealed the peripheral cellular profile of SARS-CoV-2 infection by single-cell mass cytometry and found that COVID-19 promoted T cell polarization and induced dysregulation of monocyte subpopulation homeostasis by increasing nonclassical monocytes, among others [51]. CD4^+^ T cells are widely distributed in various cancers and several studies have shown that levels of CD4^+^ T lymphocytes gradually decreases with HCC progression. Furthermore, expression level was higher in tumor tissues than in peritumor tissues, and the number of CD4^+^ T lymphocytes were significantly higher in HBV and HCV-associated HCC than in cirrhotic tissues [52, 53].

We conducted further analysis on the key modules identified from the WGCNA analysis, and identified hub genes using LASSO regression: MYLK2, FAM83D, STC2, CCDC112, EPHX4 and MMP1. MYLK2 is a calcium/calmodulin dependent enzyme, and dysregulation of MYLK2 has been detected in several types of cancer, such as pancreatic cancer and colorectal cancer [54, 55]. FAM83D is dysregulated in various tumor tissues and its upregulation in HCC is strongly associated with AJCC tumor staging, recurrence and patient survival [56]. The results of Wang et al. suggest that FAM83D may accelerate the G1-S cell cycle transition through activation of MEK/ERK signaling pathway, enhancing the growth of HCC cells [57]. STC2 expression levels are closely associated with the prognosis of HCC patients, and a dysregulated STC2 expression promotes the proliferation and metastasis of HCC cells and can lead to drug resistance [58, 59]. The CCDC family proteins contains coiled-coil structural domains that participates in various biologically functions such as cell cycle and regulation of gene expression. Changes in the structural domain of a CCDC gene or epigenetic changes are also detected in many tumors [60–62]. EPHX4 expression levels are highly upregulated in primary rectal cancer[63]. MMP1 is involved in the development of several cancers, and MMP1 upregulation promotes extracellular matrix degradation during the epithelial-mesenchymal transition and enhance migration and invasion of HCC cells [64]. Inhibition of MMP1 can impede HCC progression [65]. Our study identified that MYLK2, FAM83D, STC2, CCDC112, EPHX4 and MMP1 as significant genes for patient survival. Using these hub genes, we have developed a risk prediction model which shows a significantly higher OS rate in the low risk group compared to the high risk group. Further analysis indicated that the expression levels of ICB were generally higher in the high-risk group than the low-risk group, suggesting a potential association between tumor immune escape and the regulation of patient survival by hub genes.

We conducted a gene regulatory network analysis to identify the mechanisms of gene expression regulation. TFs play a crucial role in controlling important BP such as cell differentiation, proliferation, metabolism, immune response and the maintenance of cellular homeostasis by regulating gene transcription [66]. Similarly, miRNAs play a vital role in disease processes by adjusting the translation and stability of mRNAs at the post-transcriptional level, thereby controlling cell cycle, apoptosis and inflammatory responses [67]. TEAD4 is a DNA anchored protein of the Hippo-regulated YAP transcriptional complex, and it can promote tumorigenesis by regulating cancer stemness, metastasis and drug resistance. Several studies have shown that TEAD4 promotes HCC development in multiple ways, such as promoting Jag-1 expression to inhibit apoptosis, promoting HCC cell proliferation by regulating HNF4α and inducing chromosomal instability by binding to FOXM1 [68–70]. MYOD1, a key TF promoting muscle differentiation and related gene expression, is dysregulated in a variety of tumors. A study by Zhao study demonstrated the relationship between MYOD and multidrug resistance 1(MDR1) expression, and showed that inhibition of MYOD expression increased the sensitivity of multidrug-resistant gastric cancer cells to chemotherapeutic agents [71]. SIX4 is primarily responsible for organ development, myogenesis and neurogenesis, and elevated levels of its expressions have been associated with poor prognosis in lung, breast and colorectal cancers [72–74]. Studies from He found that elevated SIX4 expression can also promote HCC metastasis by activating the expression of YAP1 and c-MET [75]. Several studies have reported that hsa-miR-124-3p is involved in tumor development, with reduced expression levels in most cancers and a strong correlation with prognosis. A recent study confirmed that low expression of hsa-miR-124-3p promotes HCC progression through upregulation of PRAS40 expression and phosphorylation of PRAS40 [76]. Hsa-miR-17-5p is closely associated with the BP of cancer and that this regulatory role may be relevant through the PI3K/AKT and the KRAS signaling pathway [77]. Dysregulated expression of hsa-miR-17-5p was also widely observed in SARS-CoV-2 infected patients [78]. Hsa-miR-145-5p inhibits epithelial-mesenchymal transition in non-small cell lung cancer cells through a mechanism that may be related to the c-Jun N-terminal kinase signaling pathway. Furthermore, hsa-miR-145 can also regulate tumor chemoresistance, migration and invasion through epithelial-mesenchymal transition [79].

We used virtual docking to assess the potential of mefloquine, thioridazine and EINECS 250-892-2 as therapeutic agents for HCC patients infected with SARS-CoV-2. Our results indicated that these drugs can bind to multiple key targets of the virus, making them promising candidates for treatment. Mefloquine, commonly used to treat malaria, has also shown antiviral activity against coronavirus in recent studies, including anti-SARS-CoV-2 activity in several cell lines, suggesting it as an alternative to anti-COVID-19 therapy [80]. Similarly, a study by Xiao also reported the effectiveness of 20 compounds, including mefloquine and thioridazine hydrochloride, found effective in inhibiting SARS-CoV-2 [81]. On the other hand, studies of cis-Sulindac sulfide, also known as EINECS 250-892-2, have demonstrated its potential inhibition of some tumor cells, but less so for SARS-CoV-2 [82]. Based on these findings, we suggest that mefloquine and thioridazine could perhaps be potential therapeutic agents for treatment of HCC patients with COVID-19.

In conclusion, our study conducted an epigenomic analysis to investigate the relationship between SARS-CoV-2 infection and HCC patients. Our findings suggest that the co-pathogenesis between the two conditions is closely associated with immune response, specifically T cell differentiation, regulation of T cell activation and monocyte differentiation. Notably, CD4^+^ T cells and monocytes appear to play an essential role in the immunoreaction induced by both diseases. Furthermore, we identified six hub genes (MYLK2, FAM83D, STC2, CCDC112, EPHX4 and MMP1) that are strongly associated with SARS-CoV-2 infection and the prognosis of HCC patients. We also established network interactions among hub genes, TFs and miRNAs to gain insights into the mechanisms of gene expression regulation. In our study, mefloquine and thioridazine have potential therapeutic applications for treating COVID-19 in combination HCC. However, it is important to note that further experimental validation and additional case studies are needed to confirm the accuracy of our results. Overall, our research offers a novel approach to identifying the shared pathogenesis between COVID-19 and HCC through comprehensive DNA methylation analysis and suggests potential avenues for developing a cure for HCC patients infected with SARS-CoV-2.

## Conclusions

In our study, we conducted an analysis of the epigenomics in both SARS-CoV-2 infected patient and HCC patients with the aim to identify shared pathogenetic process between the two diseases. Our findings shed new light on the underlying mechanisms and potential treatment options for HCC patients who also have a SARS-CoV-2 infection.

## Supplementary Information

**Additional file 1: Figure S1.** Evaluation of the seven different kinds of immune cells in SARS-CoV-2 infection.

**Additional file 2: Figure S2.** Analyze the soft threshold power (β) of the scale-free topology model fitting index and the mean connectivity of the soft threshold power (β = 3).

**Additional file 3:** **Figure S3. **The expression of DNMT1, DNMT3A and DNMT3B in different risk group.

**Additional file 4:** **Figure S4. **Gene sequence binding site for hub genes-miRNA interaction.

**Additional file 5:** **Figure S5. **Validtion analysis of hub genes. **A** Differential methylation positions (DMPs) identified in GSE174818. **B** The location of the DMPs relative to CpG islands in GSE174818. **C** The expression of hub genes in tumor tissue and normal tissues from GSE144269. **D** The expression of hub genes in tumor tissue and normal tissues from GSE214846.

**Additional file 6:** **Figure S6. **The expression of hub genes in L-02 hepatocyte cell and human HCC cells (Huh7).

**Additional file 7:** **Figure S7.** The correlation analysis between the ICB expression and the risk scores.

**Additional file 8:** **Figure S8.** The correlation analysis between the ICB expression and the risk scores.

**Additional file 9:** **Figure S9.** Data source of TFs of the hub genes.

**Additional file 10:** **Table S1 **Top10 GO enrichment results.

**Additional file 11:** **Table S2.** qPCR primers used in this study.

## Data Availability

Data and download URLs involved in this study has been described in detail in the materials and methods section.
