# Supplementary figures and images for: Comprehensive DNA methylation profiling of COVID-19 and hepatocellular carcinoma to identify common pathogenesis and potential therapeutic targets
Source: Clin Epigenetics. 2023 Jun 12;15:100. doi: 10.1186/s13148-023-01515-8 (PMC10259366; doi:10.1186/s13148-023-01515-8)

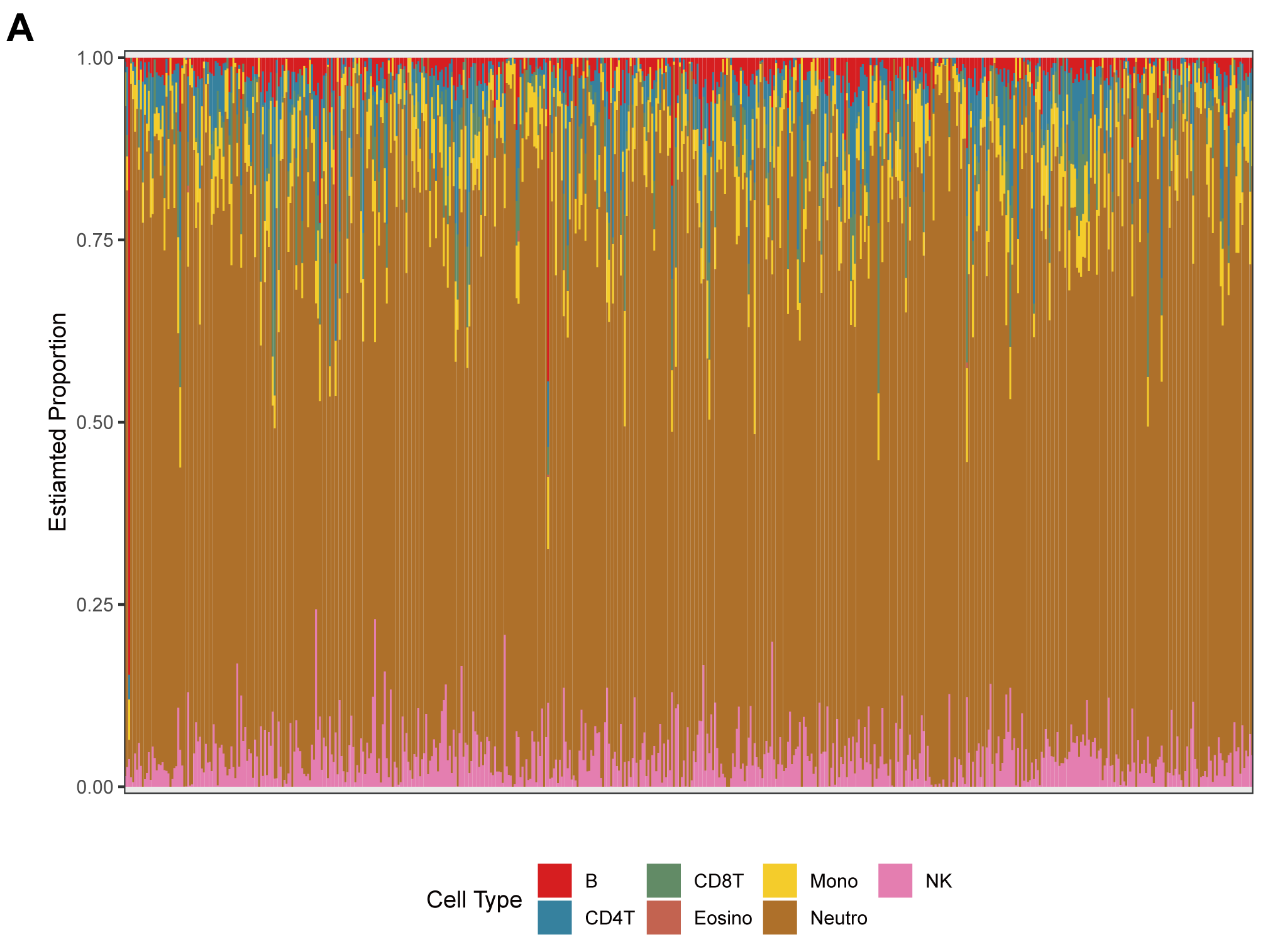

Supplement: Supplementary file 1 — Additional file 1: Figure S1. Evaluation of the seven different kinds of immune cells in SARS-CoV-2 infection. [file 13148_2023_1515_MOESM1_ESM.tif]

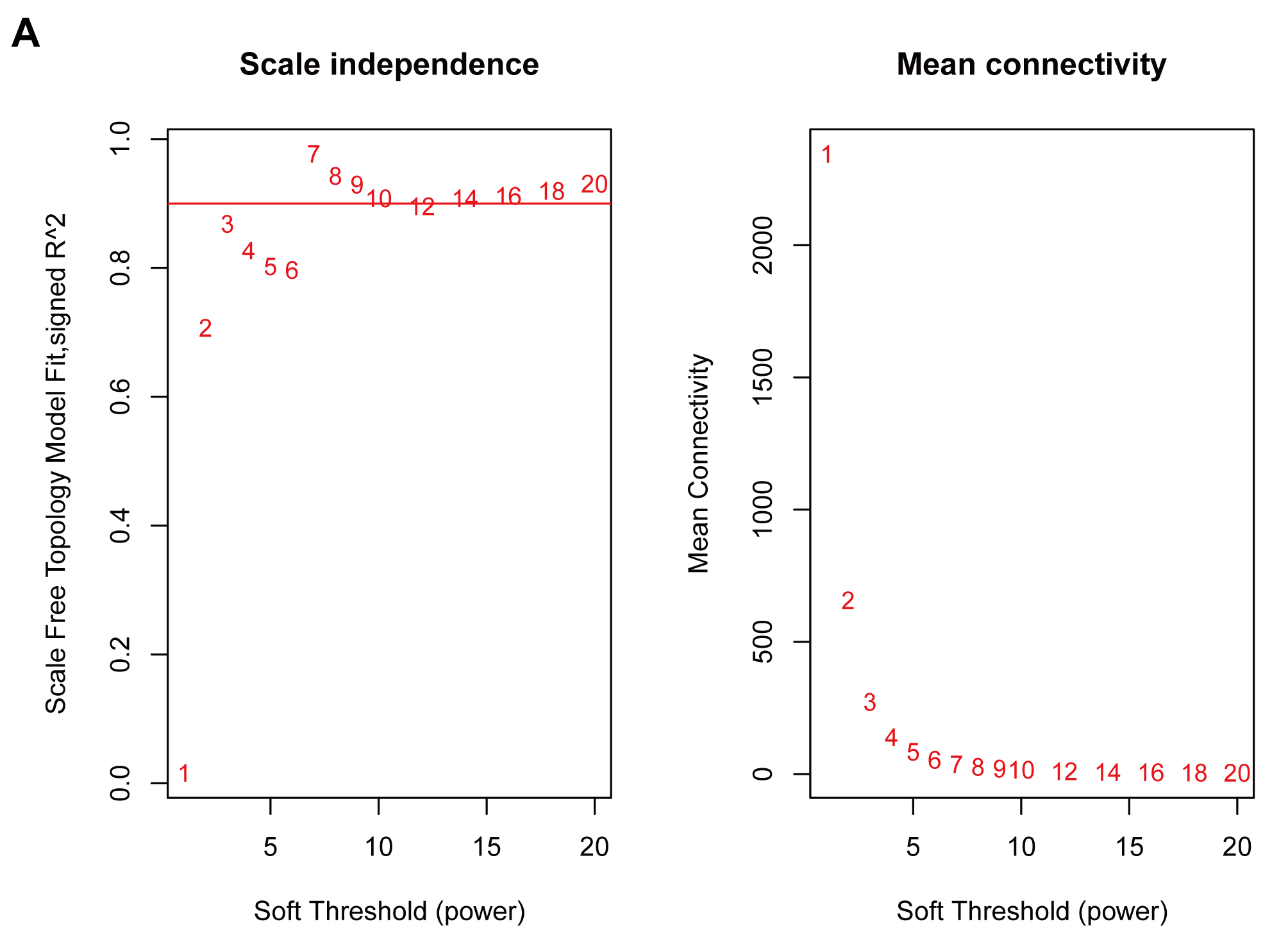

Supplement: Supplementary file 2 — Additional file 2: Figure S2. Analyze the soft threshold power (β) of the scale-free topology model fitting index and the mean connectivity of the soft threshold power (β = 3). [file 13148_2023_1515_MOESM2_ESM.tif]

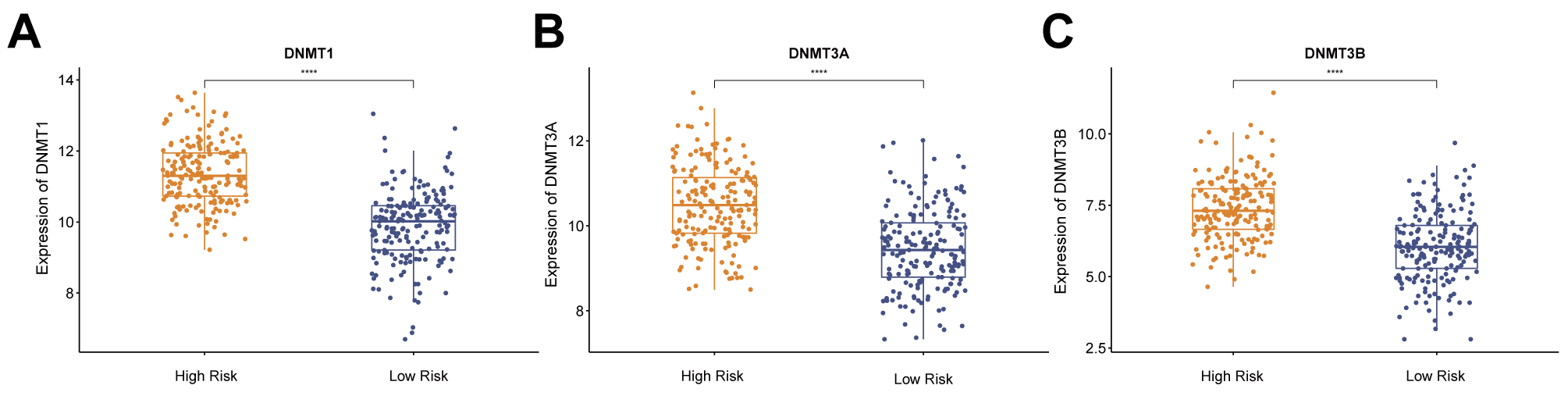

Supplement: Supplementary file 3 — Additional file 3: Figure S3. The expression of DNMT1, DNMT3A and DNMT3B in different risk group. [file 13148_2023_1515_MOESM3_ESM.tif]

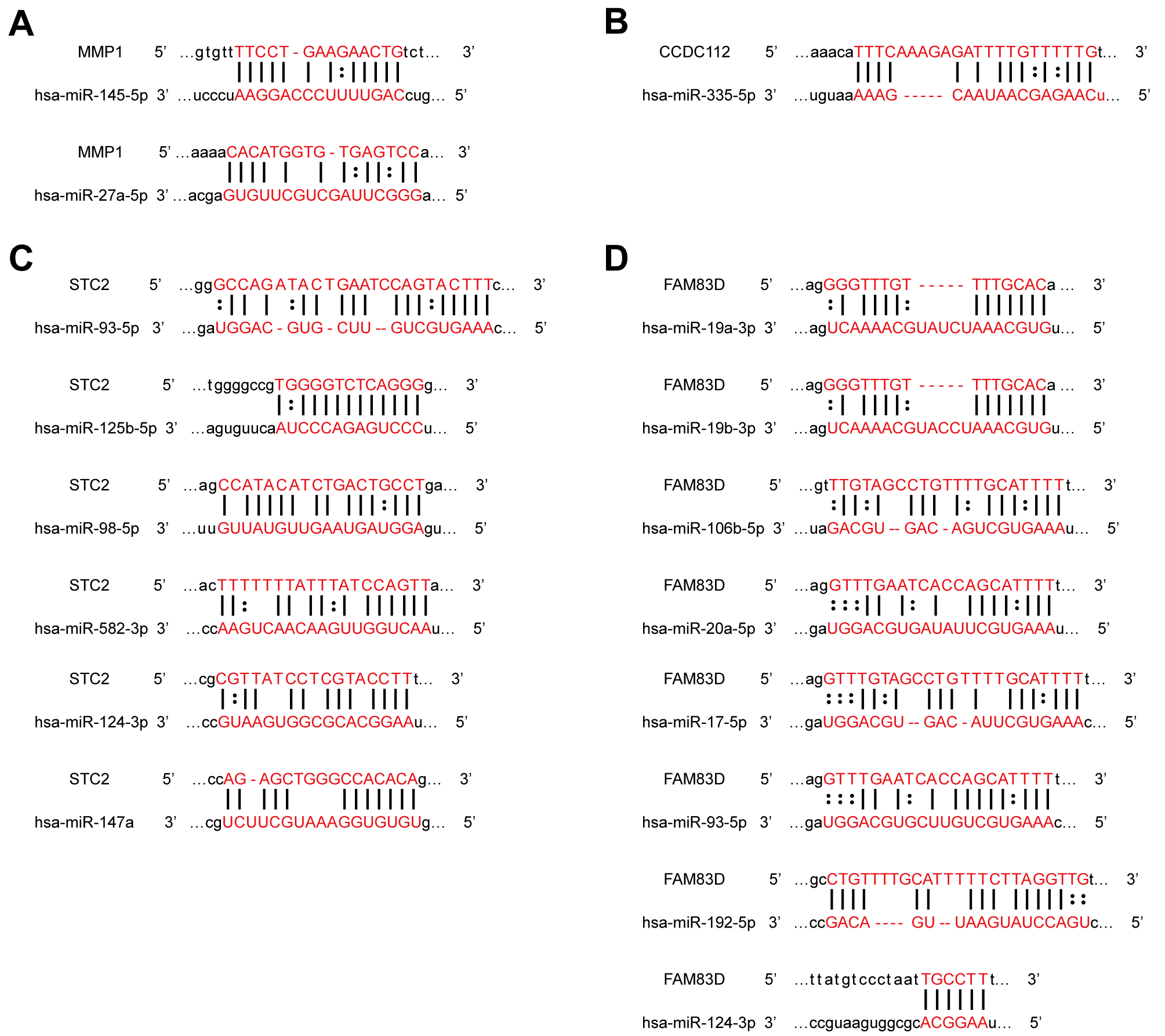

Supplement: Supplementary file 4 — Additional file 4: Figure S4. Gene sequence binding site for hub genes-miRNA interaction. [file 13148_2023_1515_MOESM4_ESM.tif]

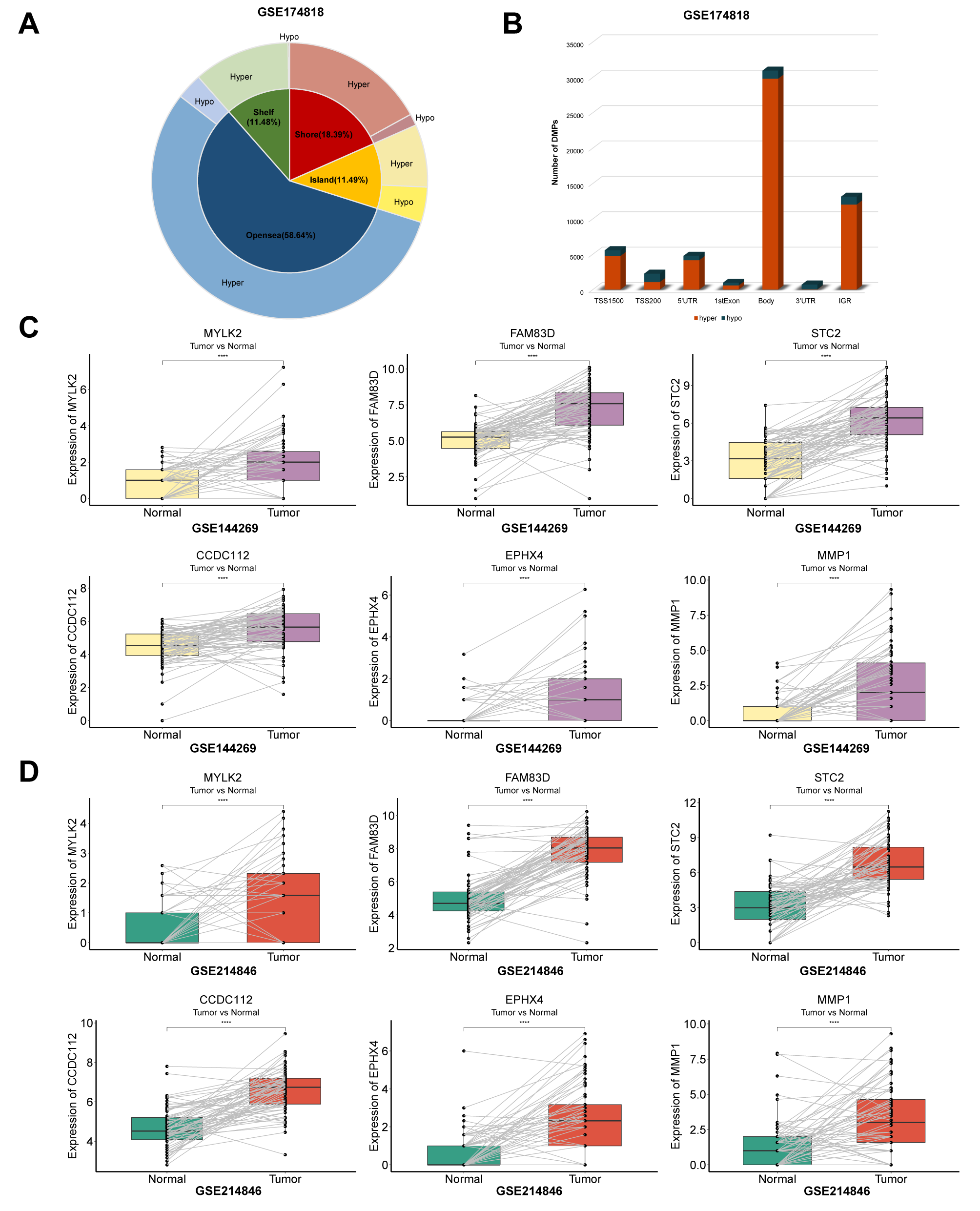

Supplement: Supplementary file 5 — Additional file 5: Figure S5. Validtion analysis of hub genes. A Differential methylation positions (DMPs) identified in GSE174818. B The location of the DMPs relative to CpG islands in GSE174818. C The expression of hub genes in tumor tissue and normal tissues from GSE144269. D The expression of hub genes in tumor tissue and normal tissues from GSE214846. [file 13148_2023_1515_MOESM5_ESM.tif]

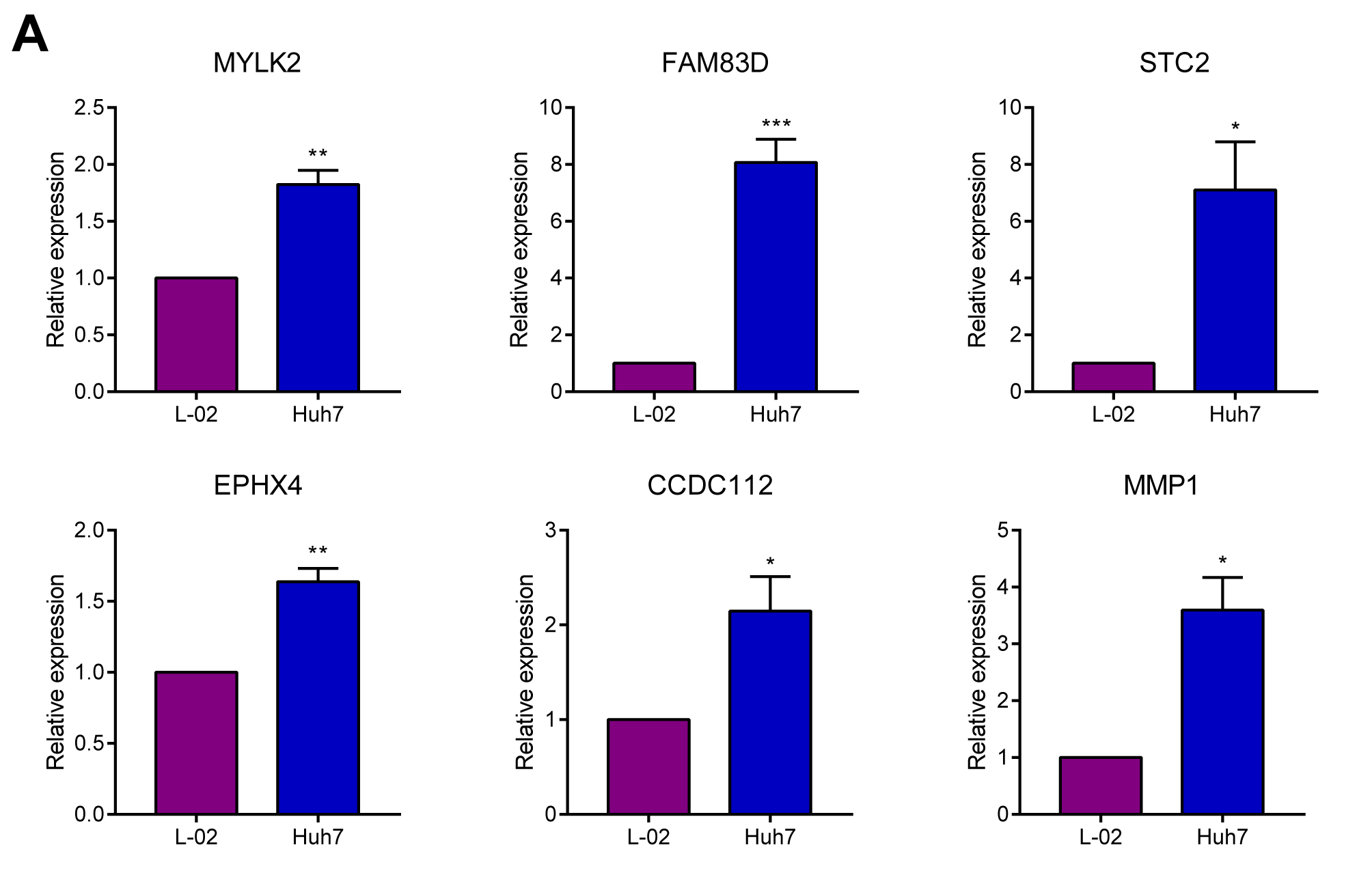

Supplement: Supplementary file 6 — Additional file 6: Figure S6. The expression of hub genes in L-02 hepatocyte cell and human HCC cells (Huh7). [file 13148_2023_1515_MOESM6_ESM.tif]

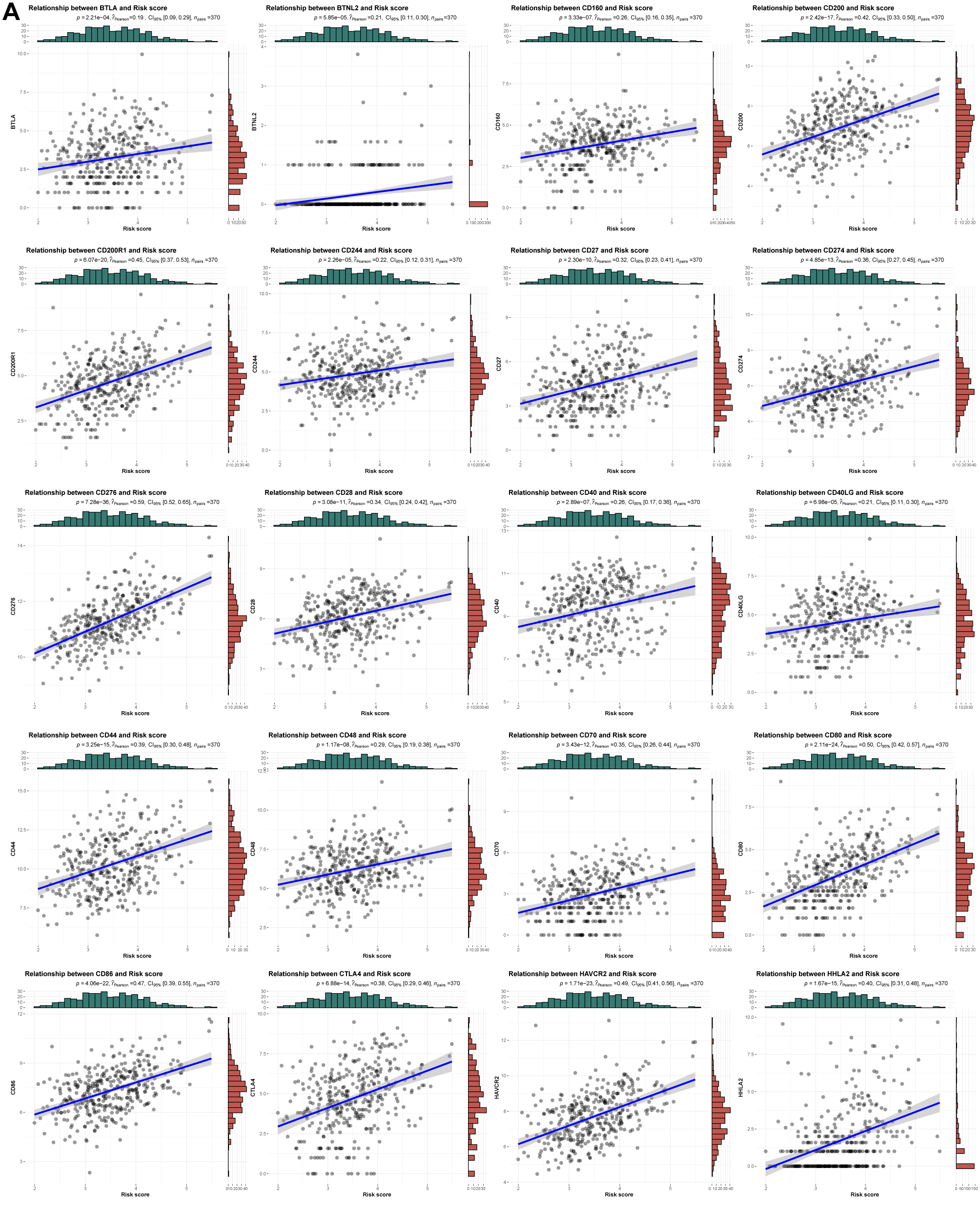

Supplement: Supplementary file 7 — Additional file 7: Figure S7. The correlation analysis between the ICB expression and the risk scores. [file 13148_2023_1515_MOESM7_ESM.tif]

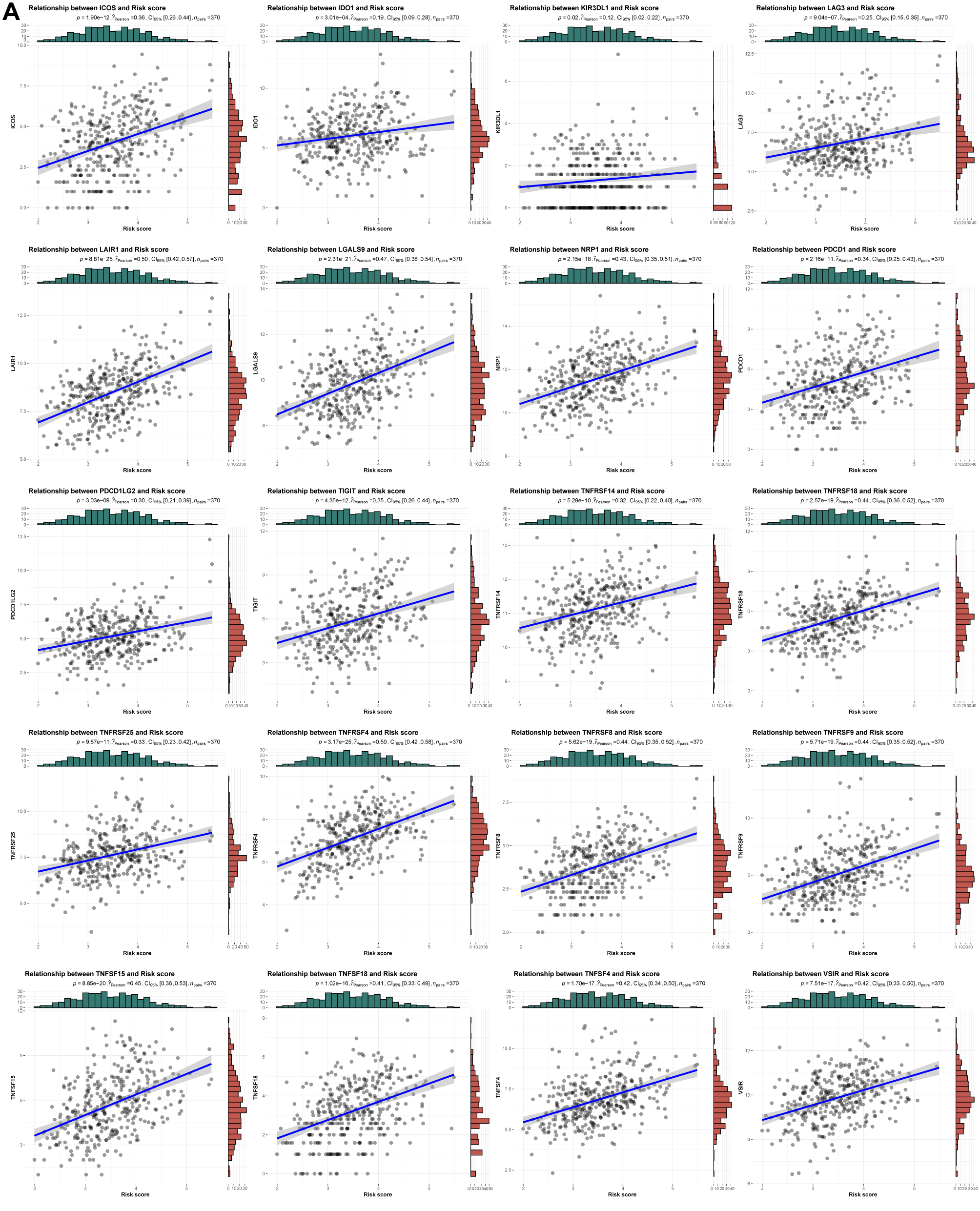

Supplement: Supplementary file 8 — Additional file 8: Figure S8. The correlation analysis between the ICB expression and the risk scores. [file 13148_2023_1515_MOESM8_ESM.tif]

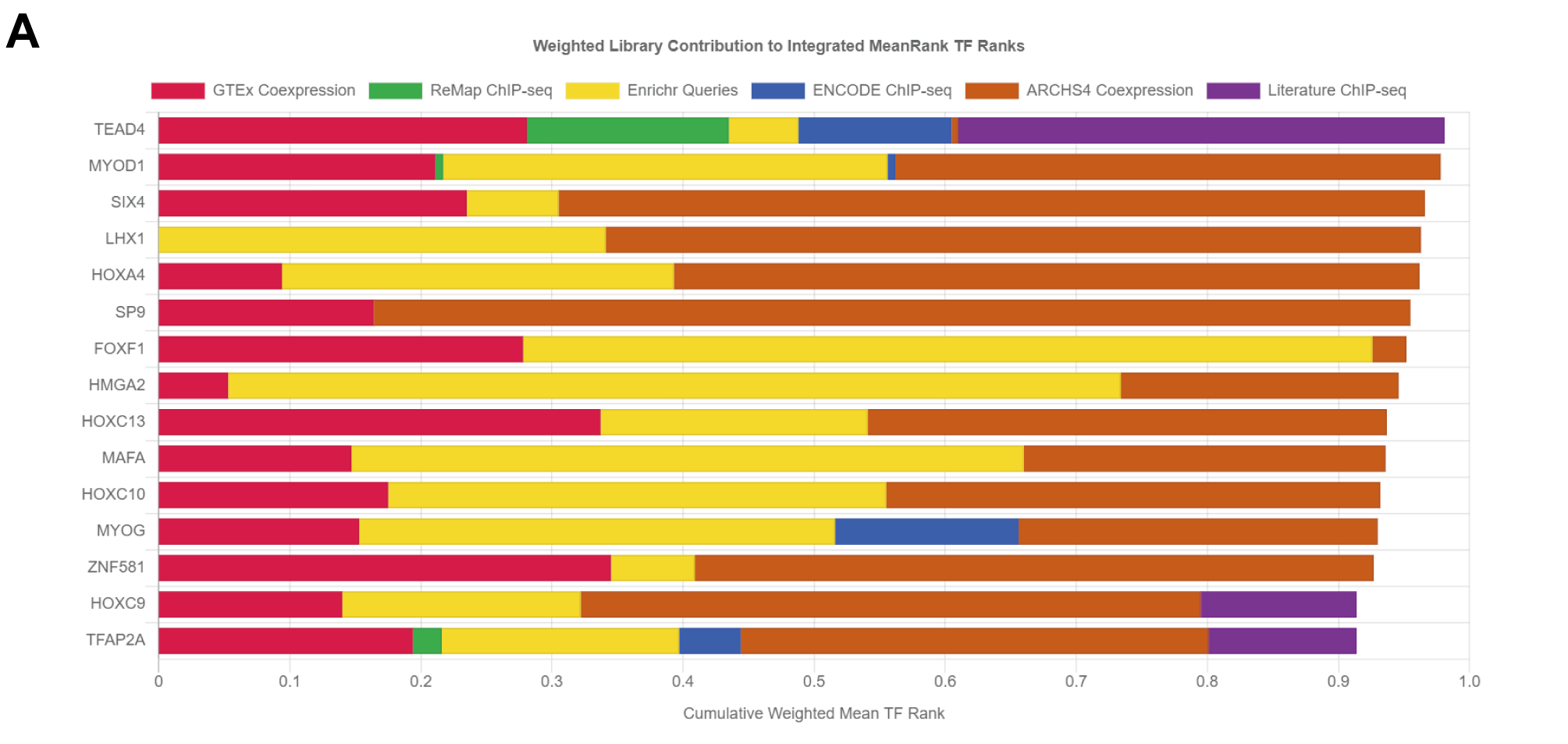

Supplement: Supplementary file 9 — Additional file 9: Figure S9. Data source of TFs of the hub genes. [file 13148_2023_1515_MOESM9_ESM.tif]
