# Supplementary material for: Comprehensive DNA methylation profiling of COVID-19 and hepatocellular carcinoma to identify common pathogenesis and potential therapeutic targets
Source: Clin Epigenetics. 2023 Jun 12;15:100. doi: 10.1186/s13148-023-01515-8 (PMC10259366; doi:10.1186/s13148-023-01515-8)
Supplement: Supplementary file 10 — Additional file 10: Table S1 Top10 GO enrichment results. [file 13148_2023_1515_MOESM10_ESM.doc]

**Supplementary Table 1. Top10 GO enrichment results.**

| Ontology | ID | Description | pvalue | p.adjust |
| --- | --- | --- | --- | --- |
| BP | GO:0022407 | regulation of cell-cell adhesion | 1.33E-10 | 4.10E-07 |
| BP | GO:0045785 | positive regulation of cell adhesion | 1.47E-10 | 4.10E-07 |
| BP | GO:0030217 | T cell differentiation | 2.27E-10 | 4.10E-07 |
| BP | GO:0006836 | neurotransmitter transport | 2.96E-10 | 4.10E-07 |
| BP | GO:0050863 | regulation of T cell activation | 3.22E-10 | 4.10E-07 |
| BP | GO:0060284 | regulation of cell development | 4.75E-10 | 5.04E-07 |
| BP | GO:0007159 | leukocyte cell-cell adhesion | 1.30E-09 | 1.18E-06 |
| BP | GO:1903131 | mononuclear cell differentiation | 2.56E-09 | 2.04E-06 |
| BP | GO:0022409 | positive regulation of cell-cell adhesion | 4.58E-09 | 3.24E-06 |
| BP | GO:0045055 | regulated exocytosis | 6.96E-09 | 4.43E-06 |
| CC | GO:0005667 | transcription regulator complex | 5.41E-10 | 4.18E-07 |
| CC | GO:0031252 | cell leading edge | 1.95E-09 | 7.55E-07 |
| CC | GO:0001726 | ruffle | 3.80E-08 | 9.78E-06 |
| CC | GO:0098793 | presynapse | 7.63E-08 | 1.47E-05 |
| CC | GO:0090575 | RNA polymerase II transcription regulator complex | 1.12E-07 | 1.73E-05 |
| CC | GO:0043292 | contractile fiber | 2.12E-07 | 2.73E-05 |
| CC | GO:0030016 | myofibril | 4.60E-07 | 5.08E-05 |
| CC | GO:0098978 | glutamatergic synapse | 6.64E-07 | 6.42E-05 |
| CC | GO:0101002 | ficolin-1-rich granule | 8.95E-07 | 7.68E-05 |
| CC | GO:0032587 | ruffle membrane | 1.23E-06 | 9.52E-05 |
| MF | GO:0106310 | protein serine kinase activity | 5.95E-10 | 6.50E-07 |
| MF | GO:0004712 | protein serine/threonine/tyrosine kinase activity | 1.06E-09 | 6.50E-07 |
| MF | GO:0004674 | protein serine/threonine kinase activity | 6.48E-09 | 2.64E-06 |
| MF | GO:0001228 | DNA-binding transcription activator activity, RNA polymerase II-specific | 4.76E-08 | 1.45E-05 |
| MF | GO:0001216 | DNA-binding transcription activator activity | 7.73E-08 | 1.84E-05 |
| MF | GO:0061629 | RNA polymerase II-specific DNA-binding transcription factor binding | 9.01E-08 | 1.84E-05 |
| MF | GO:0140297 | DNA-binding transcription factor binding | 1.24E-07 | 2.17E-05 |
| MF | GO:0030674 | protein-macromolecule adaptor activity | 1.43E-06 | 0.000218 |
| MF | GO:0046873 | metal ion transmembrane transporter activity | 3.25E-06 | 0.000442 |
| MF | GO:0003779 | actin binding | 8.92E-06 | 0.00109 |
