# Supplementary material for: Comprehensive DNA methylation profiling of COVID-19 and hepatocellular carcinoma to identify common pathogenesis and potential therapeutic targets
Source: Clin Epigenetics. 2023 Jun 12;15:100. doi: 10.1186/s13148-023-01515-8 (PMC10259366; doi:10.1186/s13148-023-01515-8)
Supplement: Supplementary file 11 — Additional file 11: Table S2. qPCR primers used in this study. [file 13148_2023_1515_MOESM11_ESM.doc]

**Supplementary Table2. qPCR primers used in this study.**

| Genes | Forward primer (5'->3') | Reverse primer (5'->3') |
| --- | --- | --- |
| MYLK2 | GCTGTTCCCTCAGAGAAATCC | GTGGCGGGCAATCATCCAA |
| FAM83D | GCCTGGCTCGTTTCCTGAA | GGAAGTGCGTCTCGACACG |
| STC2 | GCGTGCAGGTTCAGTGTGA | GGCCAGTCTCCCTACTGCT |
| CCDC112 | CCACCAAAGGTTGGGAAGAAC | TTGGAATAGCCCTATGTGGGAT |
| EPHX4 | TGCTTGGCTAATTGCCATCTG | CTGAGCAGGGTGTCGTAAAAT |
| MMP1 | GGGGCTTTGATGTACCCTAGC | TGTCACACGCTTTTGGGGTTT |
| β-Actin | GGCATCGTGATGGACTCCG | GCTGGAAGGTGGACAGCGA |
